# Supplementary material for: Long-term outcomes of anti-CD19 CAR T cell therapy in refractory myasthenia gravis: A case series
Source: Cell Rep Med. 2026 Aug 21;7(9):103000. doi: 10.1016/j.xcrm.2026.103000 (PMC13589498; doi:10.1016/j.xcrm.2026.103000)
Supplement: Supplementary file 1 [file mmc1.pdf]

Cell Reports Medicine, Volume *7*

Supplemental information

Long-term outcomes of anti-CD19 CAR T cell therapy in refractory myasthenia gravis: A case series

Tobias Hegelmaier, Jeremias Motte, Denise Walther, Eugen Feist, Alexander Duscha, Christiane Desel, Vaia Pappa, Martin Böttcher, Marvin Voigt, Melissa Sgodzai, Marwa Al-Dubai, Roland Schroers, Dominic Borie, Georg Schett, Dimitrios Mougiakakos, Ralf Gold, and Aiden Haghikia

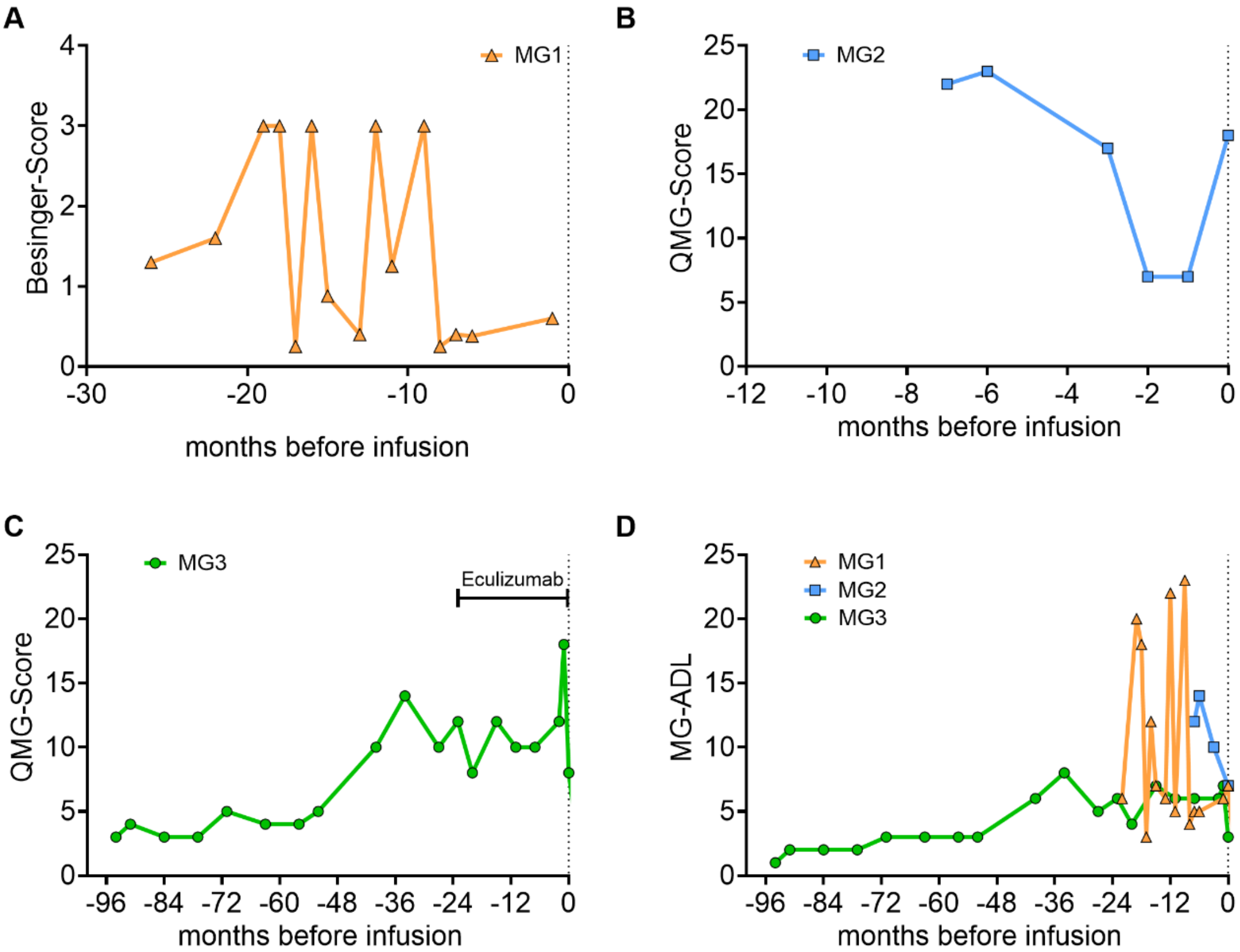

Figure S1. Baseline clinical charateristics prior to CAR T-cell infusion, Related to Tables 1-2. A: Besinger-Score of patient MG1 from March 2021 until CAR T-cell infusion in May 2023. B: Quantitative Myasthenia Gravis (QMG)-Score of patient MG2 from January 2023 until CAR T-cell infusion in August 2023. C: QMG-Score of patient MG3 from January 2016 until CAR T-cell infusion in November 2023. Eculizumab treatment from January 2022 with 900 mg escalating to 1200 mg in March 2022 until October 2023. D: Myasthenia Gravis Activities of Daily Living (MG-ADL) scores all three patients prior to CAR T-cell infusion.

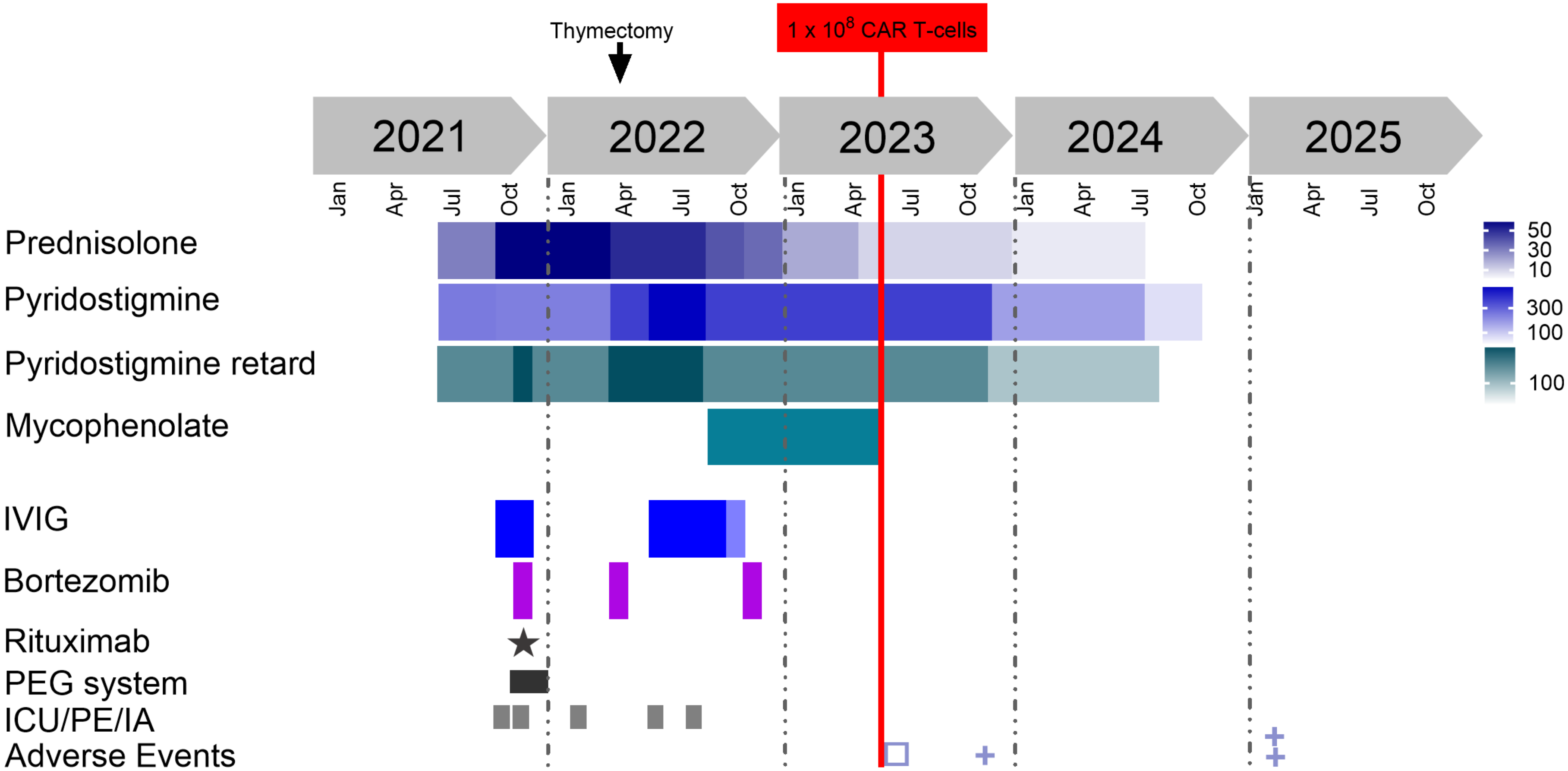

Figure S2. Treatment course of patient MG1, Related to Tables 1-2. Initial diagnosis in 2012. Doses depicted in [mg/day], unless stated otherwise. Mycophenolate: 1000 mg/day. Intravenous immune globulin (IVIG): 120 or 60 g; cumulative. Bortezomib: 4 doses each timepoint. Rituximab: 2 x 1000 mg. During acute myasthenic crisis Pyridostigmine perfusor. Intensive care unit (ICU) visits requiring intubation/ventilation. PE: plasmapheresis, IA: immunadsorption. Adverse events: Grade 1 trans-aminits (□), duration 19 days; SARS-CoV2 infection (+), duration 8 days; Pustulosis palmaris et plantaris (‡), duration 20 days; treated with Octenisept compresses (twice daily for 10–20 minutes) and local corticosteroid therapy (corticosteroid fat-based ointment, applied once daily for 4 hours).

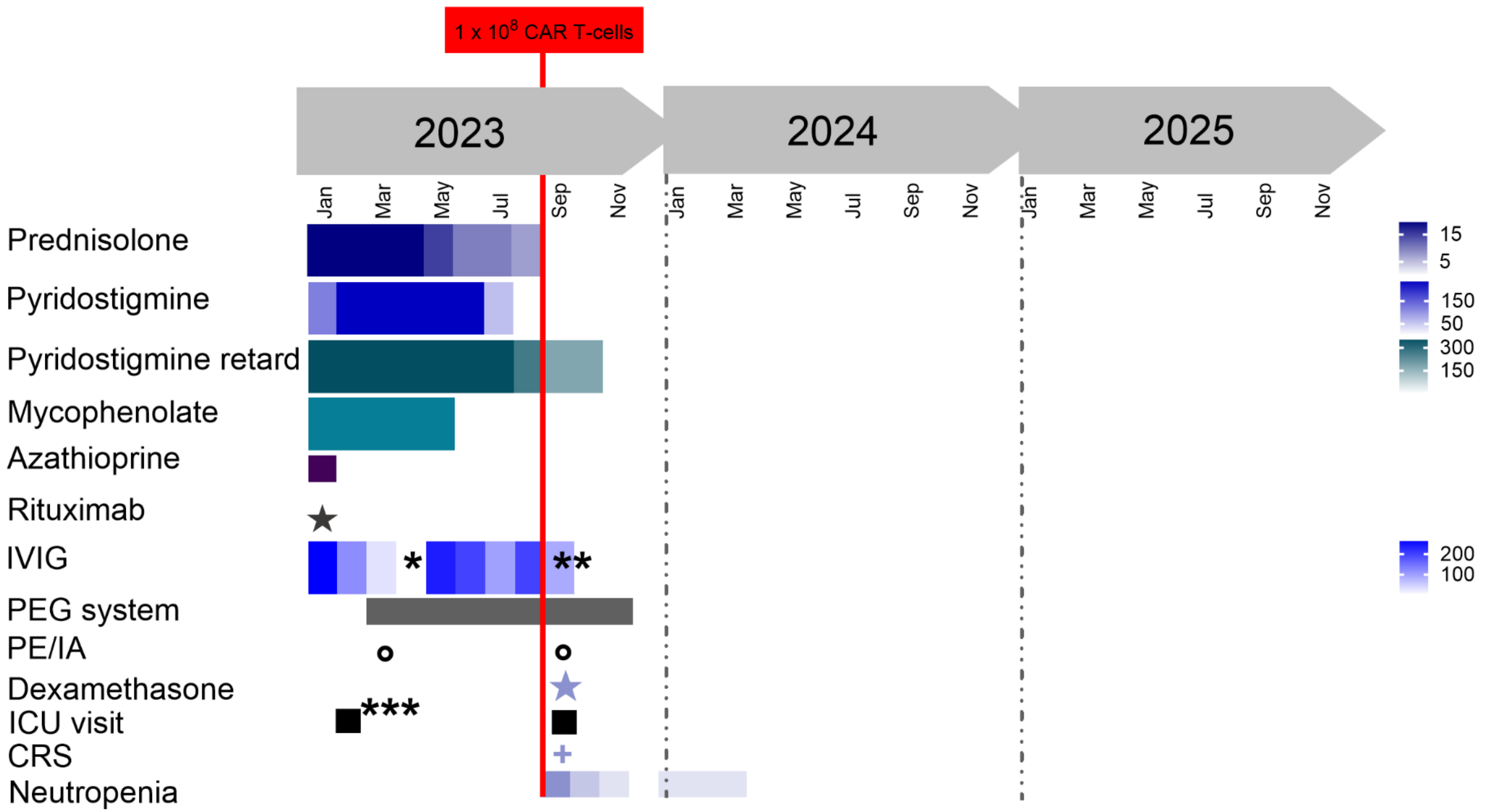

Figure S3. Treatment course of patient MG2, Related to Tables 1-2. Initial diagnosis in 2022. Doses depicted in [mg/day] unless stated otherwise. Prednisolone tapered in August 2023. During ICU visit in February 2023: 500 mg Methylprednisolone. Pyridostigmine: From July-Nov 2023 60mg daily only when necessary due to gastrointestinal adverse effects. Pyridostigmine retard: Tapered by the end of October 2023. Mycophenolate: 1000 mg. Azathioprine: Dose escalation aborted at 150mg due to side effects. Rituximab: 1 x 500 mg. Intravenous immune globulin (IVIG) in [g] cumulative dose, *April 2023: no information on IVIG dose available. ** September 2023: 90 g IVIG during ICU visit over three days. PEG: percutaneous endoscopic gastrostomy. PE: plasmapheresis, IA: immunadsorption. Dexamethasone 64 mg cumulative during ICU visit over three days. *** ICU visit for 4 days, cumulative 500mg Urbasone. ICU visits for close monitoring, not requiring intubation/ventilation. Without therapy since November 2023. Adverse events: CRS Grade 1 (+), duration 2 days; starting 6 days after CAR T-cell infusion. Grade IV neutropenia in September 2023 managed by a single dose of 32.000 IU G-CSF s.c.; recurring grade I neutropenia in 2024 lasting for 90 days without intervention.

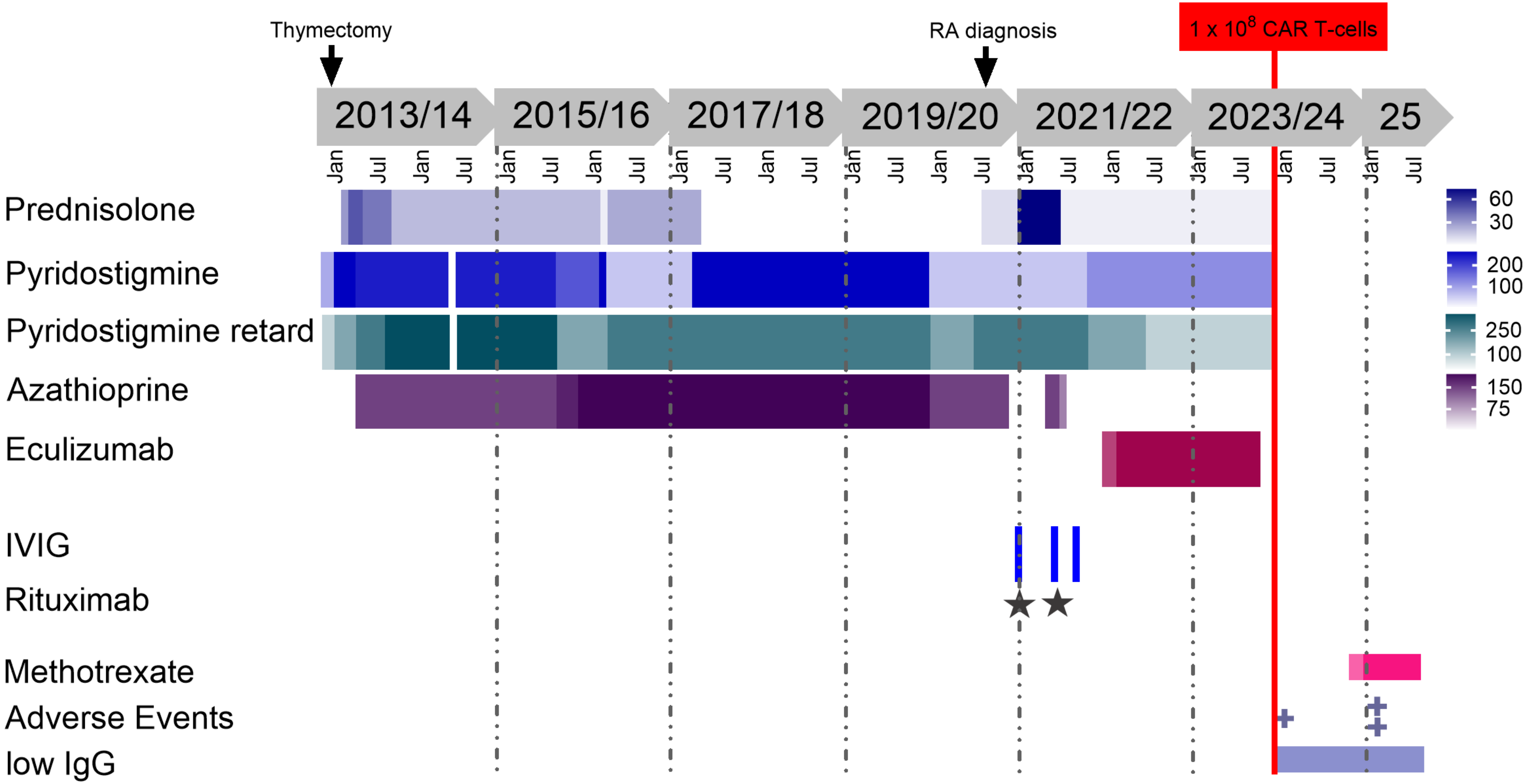

Figure S4. Treatment course of patient MG3, Related to Tables 1-2. Initial MG diagnosis in 2013, Rheumatiod arthritis (RA) diagnosis in 2020. Doses depicted in [mg/day], unless stated otherwise. Eculizumab: 900 or 1200 mg. Intravenous immune globulin (IVIG): 120 or 60 g cumulative dose. Rituximab: 500 mg. Methotrexate 10-15 mg subcutaneously. Prednisolone and Pyridostigmine were slowly tapered over the course of 5 weeks after CAR T-cell infusion. Adverse events: CRS Grade 1 (+), duration 1 day; upper respiratory tract infection (URTI) (‡). Low IgG persisting for the entire follow up period.

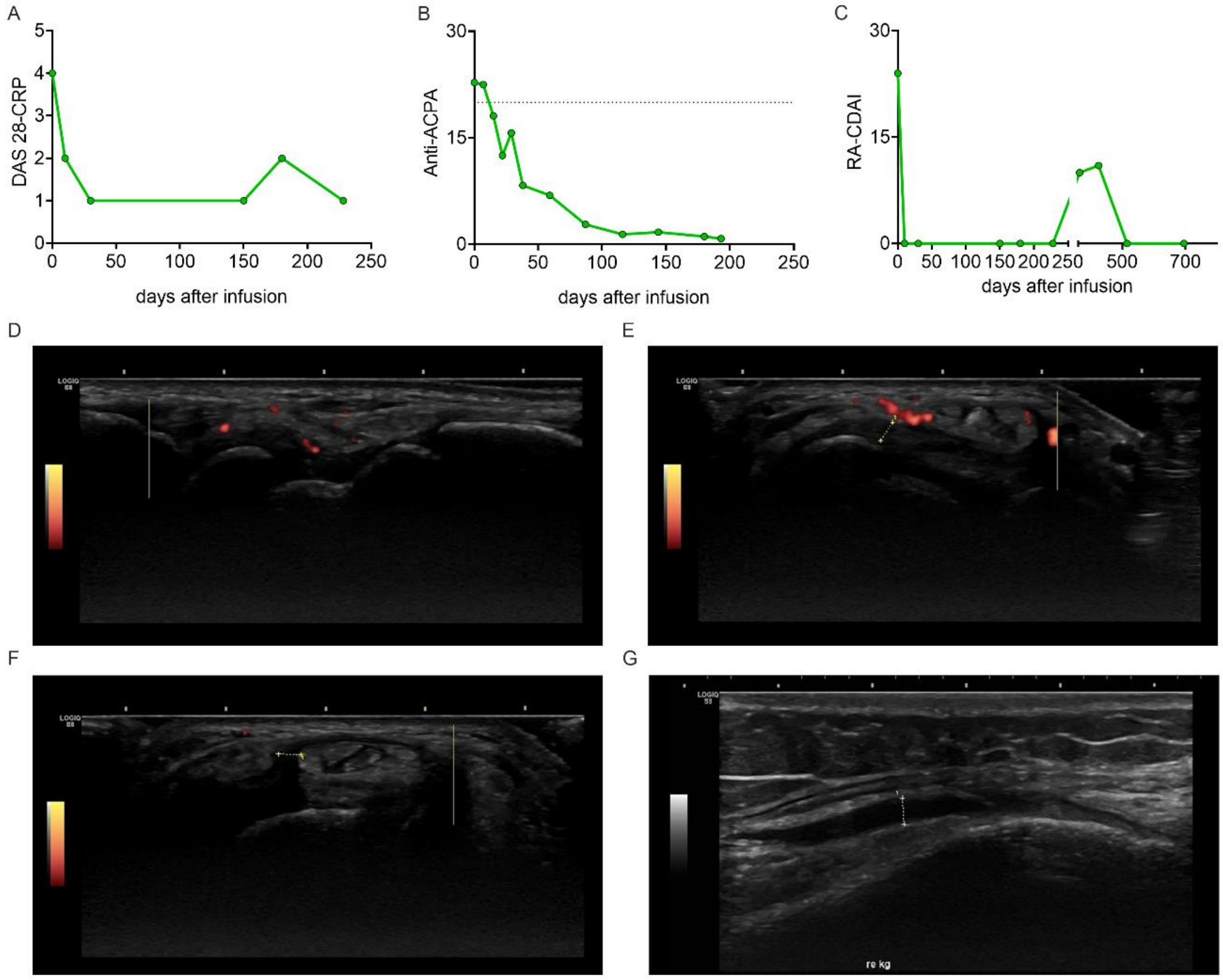

Figure S5. Serological markers of RA and ultrasound images following anti CD19 CAR T-cell therapy in patient MG3, Related to Figure 1. A: DAS-28 CRP Score. B: Anti-ACPA antibody levels. Dashed line depicts cut-off value of 20. C: RA-CDAI. D: Ultrasound image left wrist longitudinal with effusion and grade 1 synovitis. E: Ultrasound image left wrist transversal. F: Ultrasound image tenosynovitis right wrist transversal. G: Ultrasound image right knee longitudinal with mild effusion. RA = Rheumatoid Arthritis, DAS = Disease Activity Score, ACPA = Anti-Citrullinated Protein Antibodies, CDAI = Clinical Disease Activity Index.

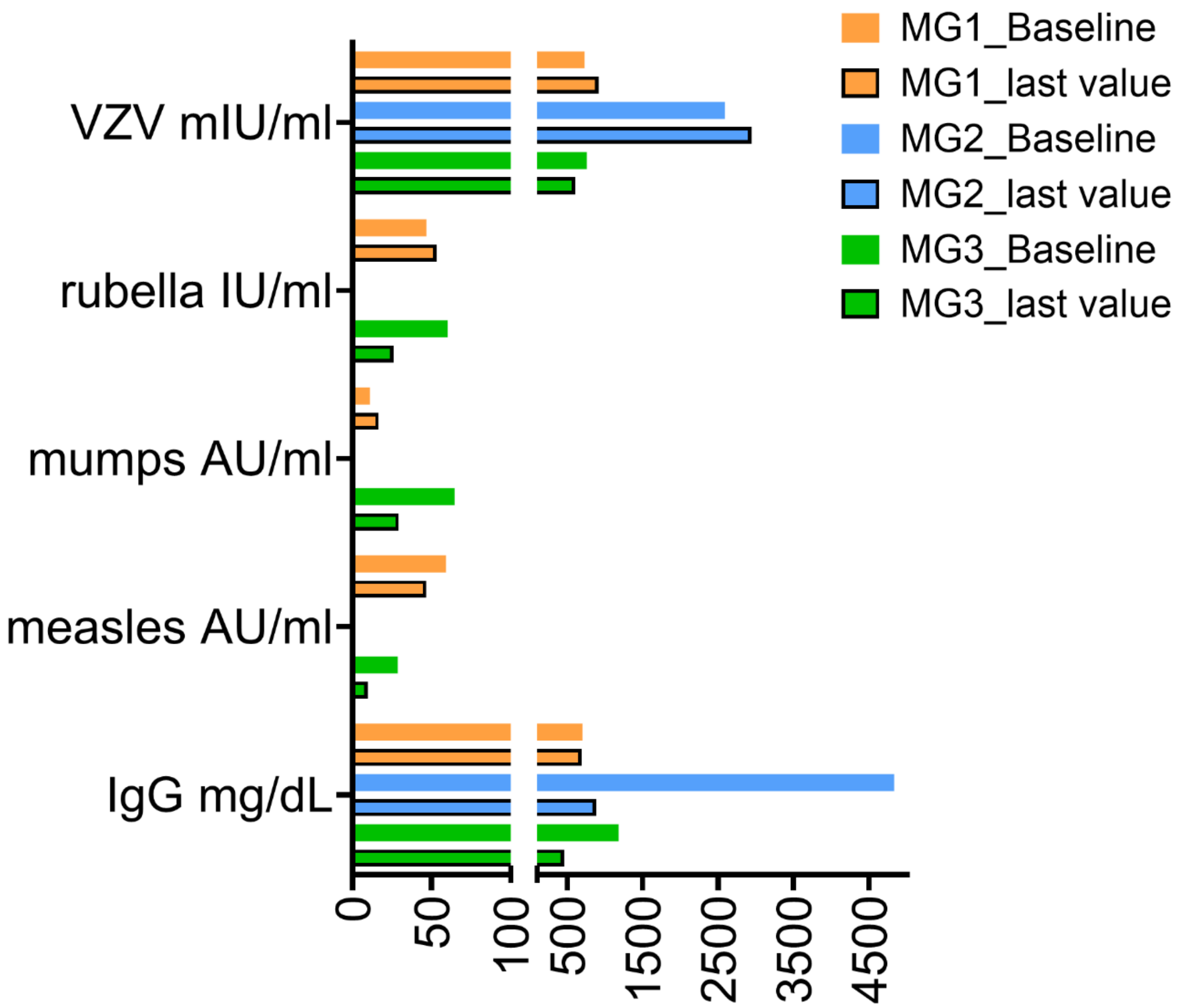

Figure S6. Vaccination titers remain stable after CAR T-cell infusion, Related to Figure 2. Baseline titers were determined before and last value refers to varying time points (60 – 200 days) after anti-CD19 CAR T-cell infusion. VZV = varicella zoster virus, MG1 = patient 1, MG2 = patient 2, MG3 = patient 3.

Panel 1 - CD4 T-cells

Cell type	Gating strategy	
**CD4+ T-cells (CD4)**	CD3^{+} CD4^{+}	
T follicular helper (Tfh)		CXCR5^{+}
T regulatory cells (Tregs)		CXCR5^{-} CD25^{high} CD127^{low}
T helper 1 (Th1)		CXCR5^{-} CD127^{+} CD25$^{-/low}$ CXCR3^{+} CCR6^{-}
T helper 1/T helper 17 (Th1/Th17)		CXCR5^{-} CD127^{+} CD25$^{-/low}$ CXCR3^{+} CCR6^{+}
T helper 17 (Th17)		CXCR5^{-} CD127^{+} CD25$^{-/low}$ CXCR3^{-} CCR6^{+}
T helper 2 (Th2)		CXCR5^{-} CD127^{+} CD25$^{-/low}$ CXCR3^{-} CCR6^{-} CCR4^{+}
T CD4 terminal effector (CD4 TE)		CXCR5^{-} CD127^{+} CD25$^{-/low}$ CXCR3^{-} CCR6^{-} CCR4^{-} CCR7^{-} CD45RA^{low}
CD4 naïve		CXCR5^{-} CD127^{+} CD25$^{-/low}$ CXCR3^{-} CCR6^{-} CCR4^{-} CCR7^{+} CD45RA^{high}

Panel 2 - CD8, γ/δ and MAIT T-cells

Cell type	Gating strategy		
CD8+ T-cells (CD8)	CD3^{+}	TCR γ/δ^{-}	CD8^{+} NOT CD161^{high} Vα7.2^{+}
γ/δ T cells		TCR γ/δ^{+}	Vd2^{+} or Vd2^{-}
MAIT		TCR γ/δ^{-} Vd2^{-}	Vα7.2^{+} CD161^{high}
CD8 naïve		CD8^{+} TCR γ/δ^{-} Vd2^{-} NOT MAIT	CCR7^{+} CD45RA^{+}
CD8 central memory T-cells (CD8 CM)			CCR7^{+} CD45RA^{-}
CD8 effector memory T-cells (CD8 EM)			CCR7^{-} CD45RA^{-}
CD8 terminal effector T-cells (CD8 TE)			CCR7^{-} CD45RA^{+}

PANEL 3 – B-cells and Progenitors

Cell type	Gating strategy		
CD34 Progenitor	CD45^{+}	CD34^{+} CD45^{low}	
Naïve B-cells (CD19 naive)		CD19^{+} CD34^{-}	CD27^{-} IgD^{+}
Nonswitched memory B-cells (CD19 NSM)			CD27^{+} IgD^{+}
Exhausted B-cells (CD19 EX)			CD27^{-} IgD^{-}
Switched memory B-cells (CD19 SM)			CD27^{+} IgD^{-} CD38^{low}
Plasmablasts			CD27^{+} IgD^{-} CD38^{high}
Plasmacells		CD19^{-} CD34^{-}	IgD^{+}

Panel 4 - Monocytes, DCs, NK cells, Low-density basophils

Cell type	Gating strategy		
NK cells	CD45^{+} CD3^{-} CD19^{-}		CD16$^{+/-}$ CD56$^{+/low}$ CD14^{-} CD11c^{-}
Classic monocytes (Mono classic)			CD14^{+} CD16^{-}
Intermediate monocytes (Mono inter)		CD11c^{+}	CD14^{+} CD16^{+}
Nonclassic monocytes (Mono nonclassic)			CD14^{low} CD16^{+}
Myeloid dendritic cells (mDC)		CD16^{-} CD56^{-}	HLA-DR^{+} CD11c^{+} CD123^{low}
Plasmacytoid dendritic cells (pDC)			HLA-DR^{+} CD11c^{-} CD123^{+}
Low-density basophils (LD basophils)			HLA-DR^{-} CD123^{+} CD11b^{+}

Figure S7. Gating strategies immunophenotyping for MG1 and MG3, Related to STAR Methods. Labelling of whole blood with fluorescent antibodies was performed according to standard protocols. Antibodies used are found in the key resource table.
