# Supplementary material for: Long-term outcomes of anti-CD19 CAR T cell therapy in refractory myasthenia gravis: A case series
Source: Cell Rep Med. 2026 Aug 21;7(9):103000. doi: 10.1016/j.xcrm.2026.103000 (PMC13589498; doi:10.1016/j.xcrm.2026.103000)
Supplement: Supplementary file 2 [file mmc2.pdf]

Cell Reports
Medicine

Report

Long-term outcomes of anti-CD19 CAR T cell therapy in refractory myasthenia gravis: A case series

Graphical abstract

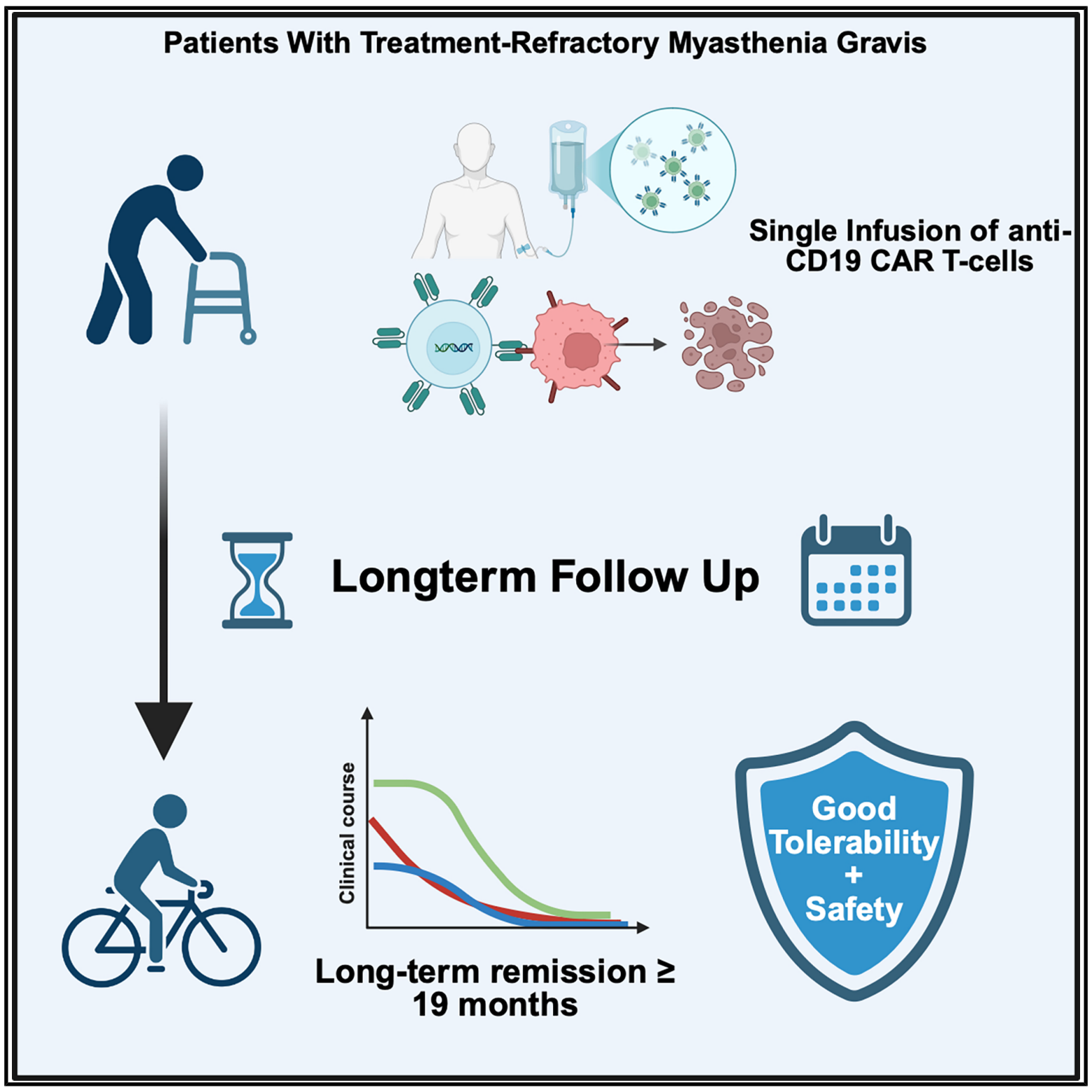

Authors

Tobias Hegelmaier, Jeremias Motte, Denise Walther, ..., Dimitrios Mougiakakos, Ralf Gold, Aiden Haghikia

Correspondence

haghikia.aiden@mh-hannover.de

In brief

Hegelmaier et al. present 2-year follow-up data from three patients with treatment-refractory myasthenia gravis receiving CAR T cells. All patients show sustained clinical remission. The long-lasting improvement in functional outcomes underscores the impact on daily living and suggests potential clinical benefit of CAR T cell therapy in selected severe neuroimmunological diseases.

Highlights

- All three patients achieve rapid, sustained clinical remission for at least 19 months
- Myasthenia gravis-specific immunotherapies can be discontinued within a few weeks
- Treatment-related adverse events are transient and manageable
- Transient B cell aplasia may induce a lasting immunological reset in autoimmunity

Hegelmaier et al., 2026, Cell Reports Medicine 7, 103000
September 15, 2026 © 2026 The Author(s). Published by Elsevier Inc.
https://doi.org/10.1016/j.xcrm.2026.103000

CellPress

Cell Reports Medicine

CellPress
OPEN ACCESS

Report

Long-term outcomes of anti-CD19 CAR T cell therapy in refractory myasthenia gravis: A case series

Tobias Hegelmaier,[1,10] Jeremias Motte,[2,10] Denise Walther,[3] Eugen Feist,[4,5] Alexander Duscha,[1] Christiane Desel,[1] Vaia Pappa,[1] Martin Böttcher,[3] Marvin Voigt,[2] Melissa Sgodzai,[2] Marwa Al-Dubai,[6] Roland Schroers,[7] Dominic Borie,[8] Georg Schett,[9] Dimitrios Mougiakakos,[3,10] Ralf Gold,[2,10] and Aiden Haghikia[1,10,11,*]

[1]Department of Neurology and Clinical Neurophysiology, Hannover Medical School, 30625 Hannover, Germany
[2]Department of Neurology, St. Josef-Hospital, Ruhr-University Bochum, 44791 Bochum, Germany
[3]Department of Hematology, Oncology, and Cell Therapy, University Hospital, Otto-von-Guericke University Magdeburg, 39120 Magdeburg, Germany
[4]Experimental Rheumatology of the Otto-von-Guericke-University Magdeburg, 39120 Magdeburg, Germany
[5]Helios Fachklinik Vogelsang-Gommern, 39245 Vogelsang-Gommern, Germany
[6]Department of Neurology, Otto-von-Guericke University Magdeburg, 39120 Magdeburg, Germany
[7]Department of Haematology and Oncology, Knappschaft Kliniken, Ruhr University Bochum, 44892 Bochum, Germany
[8]Kyverna Therapeutics, Emeryville, CA 94608, USA
[9]Department of Medicine 3, Rheumatology and Immunology, Friedrich-Alexander University, Erlangen and Universitätsklinikum Erlangen, 91054 Erlangen, Germany
[10]These authors contributed equally
[11]Lead contact
*Correspondence: haghikia.aiden@mh-hannover.de
https://doi.org/10.1016/j.xcrm.2026.103000

SUMMARY

Anti-CD19 CAR T cell therapy represents an emerging therapeutic approach for generalized, treatment-refractory myasthenia gravis (MG), a predominantly B-cell-mediated autoimmune disease for which durable treatment-free remission remains an unmet clinical goal. Despite recent advances, current targeted therapies generally require lifelong repeated administration and rarely induce durable treatment-free remission. We report on three patients with severe, treatment-resistant MG, including one patient with concomitant rheumatoid arthritis, treated with autologous, fully human anti-CD19 CAR T cells. All three patients achieve rapid, sustained clinical MG remission for at least 19 months, allowing discontinuation of MG-specific immunotherapies and substantial improvement in clinical and functional outcomes despite persistent detectable anti-AChR autoantibody titers. B cell depletion is profound, and treatment-related adverse events remain transient and manageable during long-term follow-up. These data support anti-CD19 CAR T cell therapy as a durable, effective intervention for refractory MG and warrant further evaluation in prospective controlled clinical trials.

INTRODUCTION

Myasthenia gravis (MG) is a chronic neuromuscular disorder characterized by fluctuating muscle weakness and fatigue, resulting from autoantibody-mediated impairment of acetylcholine receptors (AChRs) at the neuromuscular junction.[1] Over the past decade, significant advances have been made in the therapeutic management of this autoimmune-mediated disease,[2] and targeted immunotherapies have demonstrated clinically meaningful but overall moderate durability in randomized controlled trials. Anti-complement and neonatal Fc receptor (FcRn)-inhibiting therapies achieve Quantitative MG Score (QMG) improvements of approximately 3–5 points[3,4] and B-cell-depleting anti-CD19 antibody therapy yields comparable effects, but all require continuous administration and are associated with infection-related risks.[5] Chimeric antigen receptor (CAR) T cell technology has emerged as an effective approach for the treatment of non-solid cancer, particularly through the targeted elimination of malignant B cells, which has revolutionized outcomes for patients.[6] Anti-CD19 CAR T cell approaches result in deep and sustained B cell depletion in primary and secondary lymphoid tissues and possibly diseased organs.[7–9] The success has inspired its use beyond oncology, as autoreactive B cells have been identified to play a pivotal role in the pathogenesis of various autoimmune conditions. Especially, the potential of long-term remission is compelling[10,11] and has spurred translational approaches in neuroimmunology.[12–14] Emerging non-integrating RNA CAR T cell therapies targeting BCMA have shown early proof-of-concept efficacy, with transient but clinically relevant QMG improvements in phase 1/2 studies[15] and without significant cytokine release syndrome (CRS) or prolonged cytopenias.[16,17]

By contrast, autologous hematopoietic stem cell transplantation (aHSCT) has been associated with long-lasting, near-complete, or therapy-free remission in selected patients with severe

This is an open access article under the CC BY license (http://creativecommons.org/licenses/by/4.0/).

MG, including those with Myasthenia Gravis Foundation of America (MGFA) class IIIb–V. However, aHSCT may cause substantial short- and long-term toxicities, including febrile neutropenia, viral reactivations, secondary autoimmunity, and chemotherapy-related organ toxicity following intensive immune ablation with cyclophosphamide-based mobilization, $CD34^+$-selected autologous grafts, and antithymocyte globulin-containing conditioning regimens.[18] Nevertheless, the need for repeated administration of current immunotherapies may interfere with daily activities in a subset of patients, and some patients continue to experience clinical flare-ups, infections, or treatment-related adverse events. Achieving durable, treatment-free long-term remission remains an unmet clinical goal.

The key question remains whether anti-CD19 CAR T cell therapy can induce long-lasting remission in MG patients. KYV-101 is an autologous anti-CD19 CAR T cell therapy comprising a fully human CD19-binding domain, a CD8α hinge and transmembrane domain, a CD28 costimulatory domain, and a CD3ζ activation domain[19]; it is under investigation in B-cell-driven autoimmune diseases and one of the first fully human CD19-directed CAR T cell products translated from oncology into autoimmunity.[12] We present the 2-year follow-up data from AChR-antibody positive MG patients treated with anti-CD19 CAR T cells, offering insights into its long-term efficacy and safety.

RESULTS

Participants

All three patients fulfilled consensus criteria for treatment-refractory generalized MG defined by persistent symptoms or treatment-limiting side effects despite adequate use of corticosteroids and at least two additional immunosuppressive therapies.[20] Baseline clinical characteristics and disease course prior to CAR T cell therapy are summarized in Figure S1. The first patient (MG1), a woman, 33 years old at the time of anti-CD19 CAR T cell infusion, with anti-AChR-positive generalized MG (gMG) since 2012, developed worsening bulbar and respiratory symptoms between 2021 and 2023, including five myasthenic crises requiring intensive care and ventilation. Disease remained refractory despite thymectomy, multiple immunosuppressive therapies, and combination treatment with glucocorticoids, mycophenolate mofetil, and bortezomib after rituximab had failed (Figure S2; Table 1). Further details have previously been reported.[12]

The second patient (MG2), a 76-year-old man with anti-AChR-positive gMG, had no history of thymectomy. He experienced three myasthenic crises in the first 10 months after diagnosis, one of which required intensive care treatment. He was treated with percutaneous endoscopic gastrostomy (PEG) 5 months after diagnosis due to severe bulbar symptoms. Despite multiple pre-treatments, including high-dose intravenous immunoglobulin (IVIG), plasmapheresis, immunoadsorption, azathioprine, mycophenolate mofetil, oral corticosteroids, and rituximab, the disease remained poorly controlled. His clinical course is consistent with treatment-refractory MG, highlighting the need for alternative therapeutic strategies beyond conventional immunosuppression (Figure S3; Table 1).

The third patient (MG3), a 37-year-old woman with anti-AChR-positive gMG diagnosed in 2013, underwent thymectomy and received multiple immunosuppressive therapies but remained symptomatic consistent with treatment-refractory disease. Her final regimen of low-dose glucocorticoids, eculizumab, and pyridostigmine failed to achieve adequate disease control, with symptom worsening between infusions and recurrent mild-to-moderate infections. During an episode of acute clinical deterioration, disease stabilization was achieved with IVIGs, and plasmapheresis was, therefore, not performed. In 2020, she also developed ACPA-positive rheumatoid arthritis (RA), which could not be effectively controlled due to overlapping immunotherapeutic requirements (Figures S4 and S5; Table 1). Further details have previously been reported.[13]

Clinical effect

As previously reported, patient MG1 showed rapid clinical improvement after anti-CD19 CAR T cell infusion.[12] This improvement occurred in a gradual and sustained manner over time and was accompanied by a marked reduction in disease severity. By day 132, her QMG score had further improved to 2 (from initially 15/39), indicating sustained disease control (Figure 1A). The Myasthenia Gravis Activities of Daily Living (MG-ADL) score normalized to 0/24 within a week, and her walking distance steadily increased from 200 m to 5 km (Figures 1B and 1C). Anti-AChR antibody titers showed a pronounced initial decline from >2,000 nmol/mL and subsequently plateaued between 550 and 650 nmol/mL, despite sustained clinical remission (Figure 1D), reflecting a partial but substantial serological response in parallel with clinical improvement.

Patient MG2 showed a more gradual yet also sustained clinical improvement after anti-CD19 CAR T cell infusion (Figures 1A and 1B). Consistent with his severe initial disease course, including bulbar involvement and myasthenic crises, clinical recovery evolved progressively over several months. The PEG was removed after 3 months (Figure S3); he regained full walking ability on day 208 (Figure 1C) and could undertake mountain hikes. This clinical improvement was paralleled by a marked, approximately 10-fold reduction in anti-AChR antibody titers (Figure 1D), consistent with an expected antibody-associated response pattern in MG.

As previously reported, patient MG3 also showed rapid clinical improvement after anti-CD19 CAR T cell infusion,[13] with full walking capacity restored by day 29. Although this represents a notably faster clinical response, we acknowledge that, in the absence of contemporaneous electrophysiological confirmation, a functional overlay cannot be fully excluded; moreover, anti-AChR antibody titers remained stable, albeit substantially lower in comparison with patients MG1 and MG2 (Figure 1). This discordant response pattern contrasts with the more conventional clinical-serological coupling observed in MG1 and MG2. RA rapidly remitted, however, approximately 12 months after CAR T cell infusion, and while MG symptoms were still absent, she developed new symptoms of inflammatory arthritis involving both wrists and arthralgias of both hip joints, with a tender joint count (TJC) of 4, a swollen joint count (SJC) of 1, and a Clinical Disease Activity Index (CDAI) of 10. Ultrasound confirmed mild synovitis and tenosynovitis (Figure S5). Methotrexate 10 mg subcutaneously weekly was initiated and increased to 15 mg/week at 14 months after CAR T cell infusion

Table 1. Demographic and baseline clinical characteristics of patients prior to and at the time of anti-CD19 CAR T cell infusion

	Patient MG1	Patient MG2	Patient MG3
Age (y)	33	76	37
Sex	F	M	F
Diseases duration (y)	11	0.75	10
Follow-up (months)	24	19	19
Baseline AChR AAb titer	2,434 nmol/mL	7,020 nmol/mL	11 nmol/mL
MGFA classification	IIb	IVb	IIIa
QMG baseline	18	8	21
MG-ADL baseline	12	3	14
Walking distance baseline (m)	200	5,000	1,000
Pre-treatment			
Year of thymectomy	2022	N/A	2013
Glucocorticoids	+	+	+
Rituximab	+	+	+
Bortezomib	+	0	0
Methotrexate	0	0	0
Eculizumab	0	0	+
IVIG	+	+	+
Mycophenolate	+	+	0
Azathioprine	0	+[a]	+
PE/IA	+	+	0
Pyridostigmine	+	+	+
Pyridostigmine retarded	+	+	+
Number of ICU visits	5	1	0
Comorbidity			
Sideropenic anemia	+	0	+
Rheumatoid arthritis	0	0	+
CAR T cell parameters			
Lymphodepletion regimen	Fludarabine (30 mg/m^2)/ cyclophosphamide (300 mg/m^2) (days −6, −5, and −4)	Fludarabine (30 mg/m^2)/ cyclophosphamide (300 mg/m^2) (days −5, −4, and −3)	Fludarabine (30 mg/m^2)/ cyclophosphamide (300 mg/m^2) (days −5, −4, and −3)
Product	KYV-101	KYV-101	KYV-101
Anti-CD19 CAR T cell dose	1×10^8 cells	1×10^8 cells	1×10^8 cells

Data include age, sex, disease duration, prior therapies, baseline laboratory values, and clinical scores relevant to MG status.
Baseline parameters were recorded immediately before CAR T cell administration (day 0).
AAb, autoantibody; ICU, intensive care unit; MG-ADL, Myasthenia Gravis Activities of Daily Living; MGFA, Myasthenia Gravis Foundation of America; QMG, Quantitative Myasthenia Gravis; IVIG, intravenous immunoglobulin; PE, plasmapheresis; IA, immunoadsorption; N/A, not applicable; M, male; F, female.
[a]Dose escalation aborted at 150 mg due to side effects.See also Figures S1–S4.

due to persistent and progressive arthritis (TJC 6, SJC 0, and CDAI 11). Clinical remission was achieved by 17 months and sustained at 22 months post-infusion (TJC/SJC 0, VAS 0, and CDAI and Simplified Disease Activity Index [SDAI] 0) (Figures S4 and S5).

Safety

Patients MG1 and MG3 did not experience serious side effects (Figures S2 and S4; Table 2).[12,13] MG2 developed CRS grade 1 and grade IV neutropenia within the first 3 months (Figure S3; Table 2); the neutropenia was managed with a single administration of granulocyte colony-stimulating factor (32.000 IU); no further pharmacological interventions were required. After normalization, a recurrent grade I neutropenia occurred, which resolved spontaneously within 90 days without further intervention. No prolonged neutropenia was observed in the remaining patients (Figures S2 and S4; Table 2). Neither patient exhibited neurological adverse events (i.e., immune effector cell-associated neurotoxicity syndrome), opportunistic infections, or secondary autoimmunity during the follow-up period (Figures S2–S4; Table 2). A transient grade 1 elevation of liver enzymes (transaminitis) was observed in patient MG1 and resolved

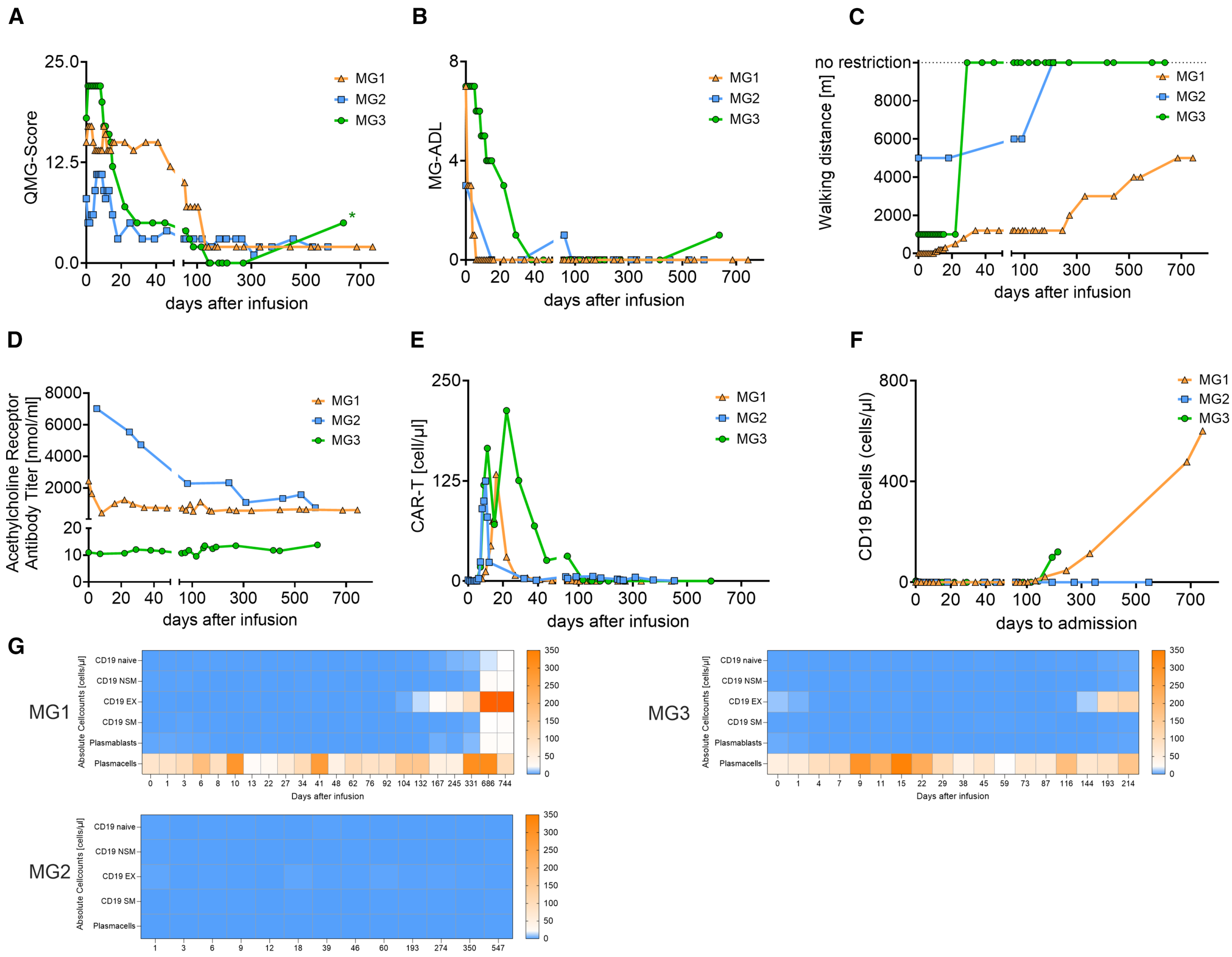

Figure 1. Longitudinal clinical and serological outcomes following anti-CD19 CAR T cell therapy

(A) Quantitative Myasthenia Gravis (QMG) scores.
(B) Myasthenia Gravis Activities of Daily Living (MG-ADL) scores.
(C) Walking distance (in meters).
(D) Serum anti-acetylcholine receptor (AChR) antibody titers (in nmol/mL).
(E) Circulating CAR T cell counts per µL blood.
(F) Circulating $CD19^{+}$ B cells per µL blood.
(G) Heatmaps depicting absolute counts of B cell subsets per µL blood. NSM, non-switched memory; EX, exhausted; SM, switched memory B cells.
All measured from the day of CAR T cell infusion (day 0). All data points represent individual patient values at the indicated time points post-infusion. MG1, patient 1; MG2, patient 2; MG3, Patient 3. *Without hand grip test and vital capacity. See also Figure S5.

without specific intervention. Infectious events were infrequent and mild. Patient MG1 developed a SARS-CoV-2 infection during follow-up, whereas patient MG3 experienced an uncomplicated upper respiratory tract infection at a later time point. Patient MG2 did not develop any infectious complications. Hypogammaglobulinemia was observed in patient MG3 and persisted over follow-up without requiring immunoglobulin replacement (Figure S4; Table 2).

CAR T cell expansion and B cell depletion

Whole-blood immunophenotyping was performed following anti-CD19 CAR T cell infusion. The kinetics of CAR T cell expansion and persistence in our patients were broadly consistent with published data in autoimmune and oncological contexts.[11,19] Patient MG1 displayed CAR T cell expansion kinetics mirroring prior lymphoma cohorts treated with the same construct and CAR T cells being detectable until day 76 (Figure 1E). In patient MG2, CAR T cells expanded rapidly and peaked on day 10, followed by low but sustained detectability until day 453. Patient MG3 exhibited a biphasic expansion profile featuring two distinct peaks, followed by sustained CAR T cell detectability until day 144 (Figure 1E).

Profound $CD19^{+}$ B cell depletion persisted through day 104 in patient MG1 and day 144 in patient MG3, and it still persisted in

Table 2. Overview of adverse events observed following anti-CD19 CAR T cell therapy

	Patient MG1	Patient MG2	Patient MG3	<3 months	<12 months	<24 months
CRS (grade)	Absent	I[a]	I[a]	X	–	–
ICANS (grade)	Absent	Absent	Absent	–	–	–
Hematologic						
Neutropenia	Absent	IV	Absent	X	–	–
Thrombocytopenia	Absent	Absent	Absent	–	–	–
Anemia	Pre-existing	Absent	Pre-existing			
Liver function	Grade 1 transaminitis[b]	Absent	Absent	X	–	–
TOC treatment	Absent	Absent	Absent	–	–	–
GLC treatment	Absent	Absent	Absent	–	–	–
Infectious						
SARS-Cov-2	Present	Absent	Absent	–	X	–
URTI	Absent	Absent	Present	–	–	X
UTI	Absent	Absent	Absent	–	–	–
Others	Pustulosis palmaris et plantaris	Absent	Absent	–	–	X
Low IgG	Absent	Absent	II[b]	X	X	X
IgG substitution	Absent	Absent	Absent	–	–	–

Occurrence of side effects within defined time intervals after CAR T cell infusion.
An "X" marks the time period during which each adverse event was documented.
Adverse events include CRS, ICANS, hematotoxicity, infections, and prolonged B cell depletion.
CRS, cytokine release syndrome; ICANS, immune effector cell-associated neurotoxicity syndrome; URTI, upper respiratory tract infection; UTI, urinary tract infection; TOC, tocilizumab; GLC, glucocorticoids.
[a]ASTCT Consensus Grading for Cytokine Release Syndrome.[21]
[b]Common Terminology Criteria for Adverse Events, v.5.See also Figures S1–S4.

patient MG2 at day 547 (Figures 1F and 1G); immunoglobulin levels were preserved and vaccine-specific antibody titers were maintained, indicating intact humoral immunity despite B cell aplasia (Figure S6). Re-emerging B cells from patients MG1 and MG3 exhibited a predominantly exhausted phenotype (Figure 1G). Notably, CD19-negative plasma cells could be detected throughout the entire observation period in patients MG1 and MG3 (Figure 1G).

Immune reconstitution

All patients demonstrated the anticipated transient reduction in total leukocyte counts at baseline due to the conditioning regimen (Figure 2).[12,13] Patients MG1 and MG3 showed similar immune reconstitution kinetics. In-depth immune profiling revealed a rapid re-emergence of classic monocytes 3–4 days after CAR T cell infusion followed by re-appearance of CD4 and CD8 T cell subsets as well as natural killer (NK) and $\gamma\delta$ T cells after 3–6 days in MG1 and 9–11 days in MG3 (Figure 2). MG2 presented slower re-population kinetics, with CD4 and CD8 T cells and NK cells re-emerging by days 12–18 and $\gamma\delta$ T cell numbers still remaining low by day 547 (Figure 2).

DISCUSSION

We report the long-term course of three individual cases of treatment-refractory, seropositive, and highly active gMG who displayed sustained treatment-free clinical remission (24 months in MG1 and 19 months in MG2 and MG3) after anti-CD19 CAR T cell therapy with KYV-101. All MG-specific immunotherapies could be discontinued within the first weeks. The long-lasting improvement in functional outcomes underscores the impact of this treatment on daily living and suggests potential clinical benefit of CAR T cell therapy in selected severe neuroimmunological diseases.

$CD19^+$ B cells were swiftly eliminated by anti-CD19 CAR T cells and reconstituted in 2 of the 3 patients within a time frame similar to oncology patients, supporting the emerging concept that transient B cell aplasia may induce lasting so-called *immunological reset* in autoimmunity.[22]

An important observation from our study is the partially discordant relationship between clinical remission and anti-AChR antibody titers. Despite long-term remission, anti-AChR levels did not normalize across individual patients, unlike in other autoimmune conditions treated with CAR T cells.[11] In patients MG1 and MG2, gradual clinical improvement was accompanied by a marked decline in anti-AChR antibody titers, whereas patient MG3 showed rapid clinical improvement despite stable, yet low, antibody levels. This clinical-serological discrepancy may reflect the underlying biology of MG, in which CD19-negative long-lived plasma cells (LLPCs) sustain autoantibody production.[23] This concept is further supported by reports of antibody disappearance only after bispecific BCMA/CD19 CAR T cell therapy.[24] Another possible explanation for this discordance is the use of anti-AChR antibody assays. In our study, a previously published anti-AChR ELISA was used externally in routine diagnostics without serial dilution. Although comparable to

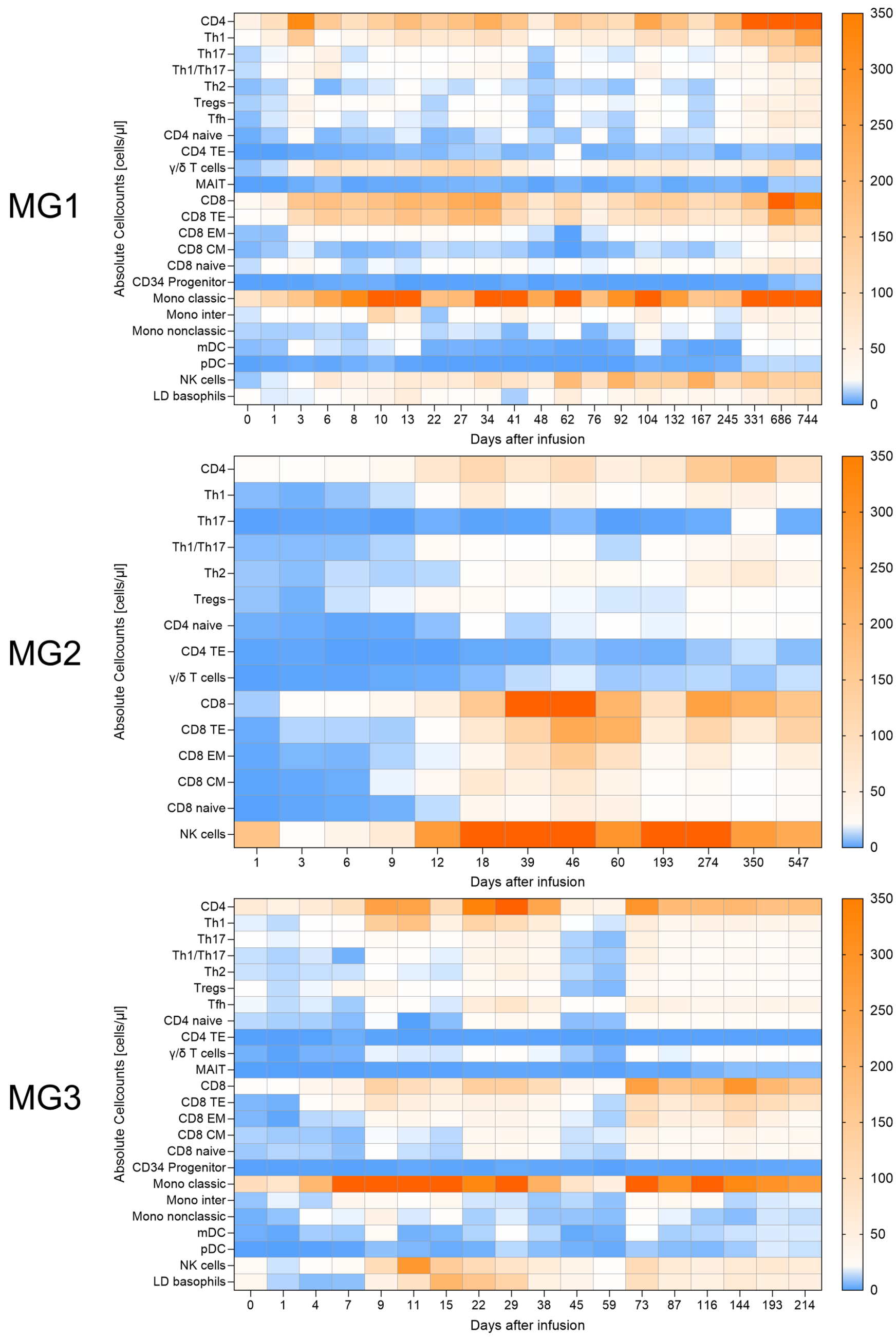

Figure 2. Absolute immune cell counts following anti-CD19 CAR T cell infusion

Heatmaps of immune cell subsets/µL blood measured by flow cytometry. CD4, CD4+ T cells; Th, CD4+ T helper cells; Tregs, CD4+ regulatory T cells; Tfh, CD4+ follicular helper cells; TE, terminal effector; MAIT, mucosal-associated invariant T cells; CD8, CD8+ T cells; EM, effector memory; CM, central memory; Mono, CD14+ monocytes; inter, intermediate; p/mDC, plasmacytoid/myeloid dendritic cells; LD, low density. Antibodies used and gating strategies are provided in the STAR Methods section. MG1, patient 1; MG2, patient 2; MG3, patient 3. See also Figure S6.

established radioimmunoassays (RIA), both methods showed variable results, especially at low antibody concentrations.[25] In addition, standard assays cannot detect low-affinity or clustered AChR-binding autoantibodies, despite their potential contribution to disease activity.[26]

In AChR antibody-positive MG, autoantibodies predominantly belong to the IgG1 and IgG3 subclasses.[27] Increasing evidence indicates substantial functional heterogeneity among AChR autoantibody populations both across patients and in response to therapy, demonstrating that pathogenicity is not determined solely by total antibody titers.[28] Accordingly, clinical remission despite persistent anti-AChR antibodies may reflect qualitative remodeling of the autoantibody repertoire, including preferential reduction of highly pathogenic subclones or shifts toward less-complement-fixing IgG fractions. Subclass-specific longitudinal data under anti-CD19 CAR T cell therapy in AChR-positive MG are currently lacking and warrant future investigation. Furthermore, immune-modulating strategies with deep tissue penetration, such as CAR T cells may clear pathogenic inflammation at the neuromuscular junction, thereby permitting structural repair.[7]

Importantly, the persistence of circulating anti-AChR antibodies argues against mechanisms relying on swift antibody clearance as the primary driver of the observed rapid clinical improvement, in contrast to therapies such as IVIG, plasmapheresis, and FcRn blockade. Instead, it may at least partially be attributed to the effects of lymphodepletion (cyclophosphamide and fludarabine), which from approximately day −6 before CAR T cell infusion markedly reduces circulating immune effector cells capable of mediating antibody-dependent inflammation.

Our data also suggest that B cells in MG contribute beyond autoantibody secretion, participating in antigen presentation and cytokine production. In MG, thymic immune dysregulation is characterized by the emergence of autoreactive CD4+ T cells, increased T follicular helper cells, and Th17 skewing, which support B cell activation and sustained autoantibody production. While such functions are generally considered to exert longer-term effects, we speculate that abrupt CD19+ B cell depletion may acutely disrupt pathogenic T cell-B cell interactions, thereby contributing to rapid functional improvement despite persistent autoantibodies.[29] This interpretation remains hypothetical and cannot be conclusively inferred from the present data, but it offers a plausible explanation consistent

with the kinetics of the clinical response observed in patient MG3.[30] Furthermore, a functional neurological overlay cannot be definitively excluded, and the relative contribution of immunological versus non-immunological factors, including potential placebo effects and prior eculizumab treatment, to the rapid recovery in MG3 requires clarification in future prospective studies incorporating electrophysiological assessments.

Patient MG3 experienced a relapse of RA about 1 year after CAR T cell therapy. This contrast in durability of response between MG and RA highlights the role of disease-specific factors and the difference in pathogenicity of the respective autoantibodies, and thus the need for tailored approaches. Mechanistically, MG is to a large extent an autoantibody-driven disease with strong dependence on $CD19^+$ B cells and plasmablast precursors, such that CD19-directed CAR T cell therapy durably disrupts the central pathogenic pathway.[31] In contrast, RA is characterized by a more complex and compartmentalized immunopathology in which autoreactive T cells, inflammatory myeloid cells, and activated synovial fibroblasts play key roles and are not directly targeted by CD19-directed CAR T cells.[32] Moreover, CD19-negative LLPCs within synovial tissue and a self-sustaining inflammatory microenvironment may permit ongoing local immune activation independent of systemic B cell depletion, predisposing to disease relapse despite persistent remission of MG.

Safety is crucial when applying CAR T cell therapy in non-malignant diseases. In our cohort, short-term adverse events were mild.[12,13] Long-term observation revealed an episode of pneumonia in patient MG3, associated with B cell aplasia and hypogammaglobulinemia. However, the overall frequency of infections was acceptable, with no life-threatening events. Recent evaluations by the European Medicines Agency identified 38 suspected cases of secondary T cell malignancies worldwide after CAR T cell therapy for malignant diseases, indicating extreme rarity; such events have not been reported in autoimmune indications to date, but warrant continued vigilance and long-term follow-up.[33] In light of the infection risk associated with all immunosuppressive treatments in patients with MG, the long-term incidence of infections appears to be lower following CAR T cell therapy, although this notion is limited by the small cohort size.[34,35] Our observations support the emerging view that anti-CD19 CAR T cell therapy represents an option to be considered in selected cases of autoimmunity, particularly those refractory to standard therapies, when compared with other immunosuppressive approaches.[36]

In conclusion, our findings support the notion that anti-CD19 CAR T cell therapy may represent a promising treatment approach for selected patients with seropositive, treatment-refractory gMG, with an acceptable safety profile and long-term remission. At the same time, interindividual differences in serological responses highlight the need for further mechanistic studies. Results support further investigation of KYV-101 in an ongoing, open-label, randomized controlled phase 2/3 study in patients with MG (NCT06193889).

Limitations of the study

A limitation of this study is that electrophysiological assessments, such as repetitive nerve stimulation or single-fiber electromyography, were not performed directly before and longitudinally after CAR T cell therapy. While all patients had shown pathological repetitive nerve stimulation at initial diagnosis, these examinations were not performed in a standardized serial manner during follow-up, which could have provided objective measures of neuromuscular transmission and may have helped to better distinguish residual myasthenic symptoms from potential functional neurological overlay, particularly in patient MG3. A further limitation of this study is the unresolved discrepancy between clinical remission and persistent anti-AChR antibody titers in patient MG3, the mechanistic basis and long-term implications of which remain unclear. Moreover, the lack of serial dilution testing of antibodies, preferably with RIA, makes these findings less reliable. In addition, the relatively small sample size and the finite follow-up period limits the generalizability of our findings.

RESOURCE AVAILABILITY

Lead contact

Further information and requests should be directed to and will be fulfilled by the lead contact, Professor Aiden Haghikia (haghikia.aiden@mh-hannover.de).

Materials availability

This study did not generate new, unique reagents.

Data and code availability

- The raw data used in the preparation of the figures and tables will be shared in an anonymized format upon reasonable request by a qualified investigator for purposes of replicating procedures and results.
- This paper does not report original code.
- Any additional information required to reanalyze the data reported in this paper is available from the lead contact upon request.

ACKNOWLEDGMENTS

We sincerely thank all patients who participated in this study for their valuable contribution. The study was supported by the Deutsche Forschungsgemeinschaft (FOR2886, RTG2408, TRR221, and Leibnitz award to G.S.). The study was supported by the European Regional Development Fund (Europäischer Fonds für Regionale Entwicklung [EFRE] project: ZELL-THEMA) to D.M. The study was supported by research funds from the state of North Rhine Westphalia, grant money from the Nasch family, and financial support from Kyverna therapeutics, Inc. to R.G.

AUTHOR CONTRIBUTIONS

Conceptualization, T.H., J.M., A.D., C.D., M.B., M.S., D.M., R.G., and A.H.; methodology, T.H., J.M., A.D., C.D., M.B., E.F., D.M., R.G., and A.H.; formal analysis, T.H., J.M., A.D., C.D., M.V., R.S., D.M., and A.H.; investigation, T.H., J.M., M.V., R.S., D.M., R.G., and A.H.; resources, T.H., J.M., R.S., D.M., R.G., and A.H.; data curation, T.H., J.M., A.D., C.D., E.F., M.V., M.S., M.A.-D., and M.S.; visualization, T.H., J.M., A.D., C.D., V.P., and M.S.; project administration, T.H., J.M., R.S., D.B., D.M., R.G., and A.H.; supervision, D.M., R.G., and A.H.; writing – original draft, T.H., A.D., C.D., and A.H.; writing – review & editing, T.H., J.M., D.W., E.F., V.P., G.S., D.M., R.G., and A.H.; validation, T.H., J.M., D.W., D.B., G.S., D.M., R.G., and A.H. All authors reviewed and approved the final manuscript.

DECLARATION OF INTERESTS

D.B. is an employee and shareholder of Kyverna Therapeutics. E.F. received honoraria from Abbvie, BMS, Galapagos, Lilly, Medac, Novartis, Pfizer, Roche, Sanofi, Sobi, and UCB. G.S. has received speaker honoraria from Bristol

Myers Squibb, Kyverna, Janssen, Miltenyi, and Novartis. R.S. has served on the scientific board of Kyverna. D.M. has received speaker honoraria and consulting fees from AbbVie, AstraZeneca, AvenCell, Bristol Myers Squibb, BeiGene, Celgene, Galapagos, Gilead, Janssen, Miltenyi, and Novartis.

STAR★METHODS

Detailed methods are provided in the online version of this paper and include the following:

- KEY RESOURCES TABLE
- EXPERIMENTAL MODEL AND STUDY PARTICIPANT DETAILS
  - Patients and clinical assessments
  - Safety assessments
  - CAR T cell manufacturing and treatment
- METHOD DETAILS
  - Immunophenotyping for MG1 and MG3
  - Immunophenotyping and CAR T cell detection for MG2
  - Detection and quantification of CAR T-cells for MG1 and MG3
- QUANTIFICATION AND STATISTICAL ANALYSIS

SUPPLEMENTAL INFORMATION

Supplemental information can be found online at https://doi.org/10.1016/j.xcrm.2026.103000.

Received: October 9, 2025
Revised: July 21, 2026
Accepted: July 24, 2026
Published: August 21, 2026

REFERENCES

1. Gilhus, N.E. (2016). Myasthenia Gravis. N. Engl. J. Med. *375*, 2570–2581. https://doi.org/10.1056/NEJMra1602678.

2. Verschuuren, J. (2023). New therapies for autoimmune myasthenia gravis. Lancet Neurol. *22*, 368–369. https://doi.org/10.1016/S1474-4422(23)00125-4.

3. Howard, J.F., Utsugisawa, K., Benatar, M., Murai, H., Barohn, R.J., Illa, I., Jacob, S., Vissing, J., Burns, T.M., Kissel, J.T., et al. (2017). Safety and efficacy of eculizumab in anti-acetylcholine receptor antibody-positive refractory generalised myasthenia gravis (REGAIN): a phase 3, randomised, double-blind, placebo-controlled, multicentre study. Lancet Neurol. *16*, 976–986. https://doi.org/10.1016/S1474-4422(17)30369-1.

4. Howard, J.F., Bril, V., Vu, T., Karam, C., Peric, S., Margania, T., Murai, H., Bilinska, M., Shakarishvili, R., Smilowski, M., et al. (2021). Safety, efficacy, and tolerability of efgartigimod in patients with generalised myasthenia gravis (ADAPT): a multicentre, randomised, placebo-controlled, phase 3 trial. Lancet Neurol. *20*, 526–536. https://doi.org/10.1016/S1474-4422(21)00159-9.

5. Nowak, R.J., Benatar, M., Ciafaloni, E., Howard, J.F., Leite, M.I., Utsugisawa, K., Vissing, J., Rojavin, M., Li, Q., Tang, F., et al. (2025). A Phase 3 Trial of Inebilizumab in Generalized Myasthenia Gravis. N. Engl. J. Med. *392*, 2309–2320. https://doi.org/10.1056/NEJMoa2501561.

6. June, C.H., O'Connor, R.S., Kawalekar, O.U., Ghassemi, S., and Milone, M.C. (2018). CAR T cell immunotherapy for human cancer. Science *359*, 1361–1365. https://doi.org/10.1126/science.aar6711.

7. Tur, C., Eckstein, M., Velden, J., Rauber, S., Bergmann, C., Auth, J., Bucci, L., Corte, G., Hagen, M., Wirsching, A., et al. (2025). CD19-CAR T-cell therapy induces deep tissue depletion of B cells. Ann. Rheum. Dis. *84*, 106–114. https://doi.org/10.1136/ard-2024-226142.

8. Hagen, M., Müller, F., Wirsching, A., Kharboutli, S., Spoerl, S., Düsing, C., Krickau, T., Metzler, M., Völkl, S., Aigner, M., et al. (2025). Local immune effector cell-associated toxicity syndrome in CAR T-cell treated patients with autoimmune disease: an observational study. Lancet Rheumatol. *7*, e424–e433. https://doi.org/10.1016/S2665-9913(25)00091-8.

9. Minopoulou, I., Wilhelm, A., Albach, F., Kleyer, A., Wiebe, E., Schallenberg, S., Fleischmann, A., Frick, M., Damm, F., Gogolok, J., et al. (2025). Anti-CD19 CAR T cell therapy induces antibody seroconversion and complete B cell depletion in the bone marrow of a therapy-refractory patient with ANCA-associated vasculitis. Ann. Rheum. Dis. *84*, e4–e7. https://doi.org/10.1016/j.ard.2025.01.008.

10. Dörner, T., and Lipsky, P.E. (2024). The essential roles of memory B cells in the pathogenesis of systemic lupus erythematosus. Nat. Rev. Rheumatol. *20*, 770–782. https://doi.org/10.1038/s41584-024-01179-5.

11. Müller, F., Taubmann, J., Bucci, L., Wilhelm, A., Bergmann, C., Völkl, S., Aigner, M., Rothe, T., Minopoulou, I., Tur, C., et al. (2024). CD19 CAR T-Cell Therapy in Autoimmune Disease - A Case Series with Follow-up. N. Engl. J. Med. *390*, 687–700. https://doi.org/10.1056/NEJMoa2308917.

12. Haghikia, A., Hegelmaier, T., Wolleschak, D., Böttcher, M., Desel, C., Borie, D., Motte, J., Schett, G., Schroers, R., Gold, R., and Mougiakakos, D. (2023). Anti-CD19 CAR T cells for refractory myasthenia gravis. Lancet Neurol. *22*, 1104–1105. https://doi.org/10.1016/S1474-4422(23)00375-7.

13. Haghikia, A., Hegelmaier, T., Wolleschak, D., Böttcher, M., Pappa, V., Motte, J., Borie, D., Gold, R., Feist, E., Schett, G., and Mougiakakos, D. (2024). Clinical efficacy and autoantibody seroconversion with CD19-CAR T cell therapy in a patient with rheumatoid arthritis and coexisting myasthenia gravis. Ann. Rheum. Dis. *83*, 1597–1598. https://doi.org/10.1136/ard-2024-226017.

14. Motte, J., Sgodzai, M., Schneider-Gold, C., Steckel, N., Mika, T., Hegelmaier, T., Borie, D., Haghikia, A., Mougiakakos, D., Schroers, R., and Gold, R. (2024). Treatment of concomitant myasthenia gravis and Lambert-Eaton myasthenic syndrome with autologous CD19-targeted CAR T cells. Neuron *112*, 1757–1763.e2. https://doi.org/10.1016/j.neuron.2024.04.014.

15. Chahin, N., Sahagian, G., Feinberg, M.H., Stewart, C.A., Jewell, C.M., Kurtoglu, M., Miljković, M.D., Vu, T., Mozaffar, T., and Howard, J.F.; MG-001 Study Group (2025). Durability of Response to B-Cell Maturation Antigen-Directed mRNA Cell Therapy in Myasthenia Gravis. Ann. Clin. Transl. Neurol. *12*, 2358–2366. https://doi.org/10.1002/acn3.70167.

16. Granit, V., Benatar, M., Kurtoglu, M., Miljković, M.D., Chahin, N., Sahagian, G., Feinberg, M.H., Slansky, A., Vu, T., Jewell, C.M., et al. (2023). Safety and clinical activity of autologous RNA chimeric antigen receptor T-cell therapy in myasthenia gravis (MG-001): a prospective, multicentre, open-label, non-randomised phase 1b/2a study. Lancet Neurol. *22*, 578–590. https://doi.org/10.1016/S1474-4422(23)00194-1.

17. Vu, T., Durmus, H., Rivner, M., Shroff, S., Ragole, T., Myers, B., Pasnoor, M., Small, G., Karam, C., Vullaganti, M., et al. (2026). BCMA-directed mRNA CAR T cell therapy for myasthenia gravis: a randomized, double-blind, placebo-controlled phase 2b trial. Nat. Med. *32*, 1131–1141. https://doi.org/10.1038/s41591-025-04171-y.

18. Bryant, A., Atkins, H., Pringle, C.E., Allan, D., Anstee, G., Bence-Bruckler, I., Hamelin, L., Hodgins, M., Hopkins, H., Huebsch, L., et al. (2016). Myasthenia Gravis Treated With Autologous Hematopoietic Stem Cell Transplantation. JAMA Neurol. *73*, 652–658. https://doi.org/10.1001/jamaneurol.2016.0113.

19. Brudno, J.N., Lam, N., Vanasse, D., Shen, Y.-W., Rose, J.J., Rossi, J., Xue, A., Bot, A., Scholler, N., Mikkilineni, L., et al. (2020). Safety and feasibility of anti-CD19 CAR T cells with fully human binding domains in patients with B-cell lymphoma. Nat. Med. *26*, 270–280. https://doi.org/10.1038/s41591-019-0737-3.

20. Sanders, D.B., Wolfe, G.I., Benatar, M., Evoli, A., Gilhus, N.E., Illa, I., Kuntz, N., Massey, J.M., Melms, A., Murai, H., et al. (2016). International consensus guidance for management of myasthenia gravis: Executive summary. Neurology *87*, 419–425. https://doi.org/10.1212/WNL.0000000000002790.

21. Lee, D.W., Santomasso, B.D., Locke, F.L., Ghobadi, A., Turtle, C.J., Brudno, J.N., Maus, M.V., Park, J.H., Mead, E., Pavletic, S., et al. (2019). ASTCT Consensus Grading for Cytokine Release Syndrome and Neurologic Toxicity Associated with Immune Effector Cells. Biol. Blood

Marrow Transplant. *25*, 625–638. https://doi.org/10.1016/j.bbmt.2018.12.758.

22. Cappell, K.M., Sherry, R.M., Yang, J.C., Goff, S.L., Vanasse, D.A., McIntyre, L., Rosenberg, S.A., and Kochenderfer, J.N. (2020). Long-Term Follow-Up of Anti-CD19 Chimeric Antigen Receptor T-Cell Therapy. J. Clin. Oncol. *38*, 3805–3815. https://doi.org/10.1200/JCO.20.01467.

23. Yi, J.S., Guptill, J.T., Stathopoulos, P., Nowak, R.J., and O'Connor, K.C. (2018). B cells in the pathophysiology of myasthenia gravis. Muscle Nerve *57*, 172–184. https://doi.org/10.1002/mus.25973.

24. Zhang, Y., Liu, D., Zhang, Z., Huang, X., Cao, J., Wang, G., Du, X., Wang, Z., Yang, M., Luo, T., et al. (2024). Bispecific BCMA/CD19 targeted CAR-T cell therapy forces sustained disappearance of symptoms and anti-acetylcholine receptor antibodies in refractory myasthenia gravis: a case report. J. Neurol. *271*, 4655–4659. https://doi.org/10.1007/s00415-024-12367-4.

25. Kawaguchi, N., Imafuku, A., Fujii, S., and Sohn, J. (2024). Performance evaluation of the EUROIMMUN anti-acetylcholine receptor enzyme-linked immunoassay. Clin. Exp. Neuroimmunol. *15*, 109–115. https://doi.org/10.1111/cen3.12774.

26. Meriggioli, M.N., and Sanders, D.B. (2012). Muscle autoantibodies in myasthenia gravis: beyond diagnosis? Expert Rev. Clin. Immunol. *8*, 427–438. https://doi.org/10.1586/eci.12.34.

27. Gilhus, N.E., Tzartos, S., Evoli, A., Palace, J., Burns, T.M., and Verschuuren, J.J.G.M. (2019). Myasthenia gravis. Nat. Rev. Dis. Primers *5*, 30. https://doi.org/10.1038/s41572-019-0079-y.

28. Khani-Habibabadi, F., Roy, B., Pham, M.C., Obaid, A.H., Filipek, B., Nowak, R.J., and O'Connor, K.C. (2025). AChR Autoantibody Pathogenic Properties Are Heterogeneously Distributed and Undergo Temporal Changes Among Patients With Myasthenia Gravis. Neurol. Neuroimmunol. Neuroinflamm. *12*, e200436. https://doi.org/10.1212/NXI.0000000000200436.

29. Huijbers, M.G., Marx, A., Plomp, J.J., Le Panse, R., and Phillips, W.D. (2022). Advances in the understanding of disease mechanisms of autoimmune neuromuscular junction disorders. Lancet Neurol. *21*, 163–175. https://doi.org/10.1016/S1474-4422(21)00357-4.

30. Samiea, A., Celis, G., Yadav, R., Rodda, L.B., and Moreau, J.M. (2025). B cells in non-lymphoid tissues. Nat. Rev. Immunol. *25*, 483–496. https://doi.org/10.1038/s41577-025-01137-6.

31. Iorio, R. (2024). Myasthenia gravis: the changing treatment landscape in the era of molecular therapies. Nat. Rev. Neurol. *20*, 84–98. https://doi.org/10.1038/s41582-023-00916-w.

32. Alivernini, S., Firestein, G.S., and McInnes, I.B. (2022). The pathogenesis of rheumatoid arthritis. Immunity *55*, 2255–2270. https://doi.org/10.1016/j.immuni.2022.11.009.

33. Berg, P., Bakker, C., Sander, M., Hasselblad Lundstrøm, N., Erneholm, K., Musuamba Tshinanu, F., Kholmanshikh, O., Van Nuffel, F., Müller, S., Ruppert-Seipp, G., et al. (2026). Secondary malignancy of T-cell origin after CAR T-cell therapy: EMA's conclusions from the evaluation of 38 suspected cases. Gene Ther. *33*, 260–265. https://doi.org/10.1038/s41434-025-00586-x.

34. Cappell, K.M., and Kochenderfer, J.N. (2023). Long-term outcomes following CAR T cell therapy: what we know so far. Nat. Rev. Clin. Oncol. *20*, 359–371. https://doi.org/10.1038/s41571-023-00754-1.

35. Clifford, K., McKeen, W., Yuan, Y., and Muppidi, S. (2023). Risk of Infection in Patients with Myasthenia Gravis With and Without Thymoma (P2-8.008). Neurology *100*, 2826. https://doi.org/10.1212/WNL.0000000000202824.

36. Kassardjian, C.D., Widdifield, J., Paterson, J.M., Kopp, A., Nagamuthu, C., Barnett, C., Tu, K., and Breiner, A. (2020). Serious infections in patients with myasthenia gravis: population-based cohort study. Eur. J. Neurol. *27*, 702–708. https://doi.org/10.1111/ene.14153.

37. Jaretzki, A., Barohn, R.J., Ernstoff, R.M., Kaminski, H.J., Keesey, J.C., Penn, A.S., and Sanders, D.B. (2000). Myasthenia gravis: recommendations for clinical research standards. Neurology *55*, 16–23. https://doi.org/10.1212/wnl.55.1.16.

38. van Gestel, A.M., Prevoo, M.L., van 't Hof, M.A., van Rijswijk, M.H., van de Putte, L.B., and van Riel, P.L. (1996). Development and validation of the European League Against Rheumatism response criteria for rheumatoid arthritis. Comparison with the preliminary American College of Rheumatology and the World Health Organization/International League Against Rheumatism Criteria. Arthritis Rheum. *39*, 34–40. https://doi.org/10.1002/art.1780390105.

39. Mougiakakos, D., Sengupta, R., Gold, R., Schroers, R., Haghikia, A., Lorente, M., Pendleton, M., Register, A., Heesen, C., Kröger, N., et al. (2025). Successful generation of fully human, second generation, anti-CD19 CAR T cells for clinical use in patients with diverse autoimmune disorders. Cytotherapy *27*, 236–246. https://doi.org/10.1016/j.jcyt.2024.09.008.

40. Monaco, G., Lee, B., Xu, W., Mustafah, S., Hwang, Y.Y., Carré, C., Burdin, N., Visan, L., Ceccarelli, M., Poidinger, M., et al. (2019). RNA-Seq Signatures Normalized by mRNA Abundance Allow Absolute Deconvolution of Human Immune Cell Types. Cell Rep. *26*, 1627–1640.e7. https://doi.org/10.1016/j.celrep.2019.01.041.

STAR★METHODS

KEY RESOURCES TABLE

REAGENT or RESOURCE	SOURCE	IDENTIFIER
Antibodies		
CD194 (CCR4), BV 421, Clone L2A1H4	BioLegend, San Diego, USA	AB_2562435
CCR6, PE, Clone 11A9	BD Biosciences, Franklin Lakes, USA	AB_397273
CD197 (CCR7), PerCP/Cy5.5, Clone G043H7	BioLegend, San Diego, USA	AB_10916121
CD11b, PE, Clone ICRF44	BioLegend, San Diego, USA	AB_314158
CD11c, APC, Clone B-LY6	BD Biosciences, Franklin Lakes, USA	AB_398680
CD123, BV605, Clone 6H6	BioLegend, San Diego, USA	AB_2563826
CD127, APC, Clone A019DS	BioLegend, San Diego, USA	AB_10900804
CD14, PE/Dazzle 594, Clone HCD14	BioLegend, San Diego, USA	AB_2563625
CD16, APC Fire 750, Clone 3G8	BioLegend, San Diego, USA	AB_2616707
CD161, PE, Clone HP3G10	BioLegend, San Diego, USA	AB_1501083
CD19, FITC, Clone H1B19	BioLegend, San Diego, USA	AB_314236
CD25, BV785, Clone M-A251	BioLegend, San Diego, USA	AB_2750205
CD27, BV421, Clone 323	BioLegend, San Diego, USA	AB_11150782
CD3, FITC, Clone UCHT1	BioLegend, San Diego, USA	AB_2562046
CD34, PE, Clone 563	BD Biosciences, Franklin Lakes, USA	AB_393871
CD38, APC, Clone HIT2	BioLegend, San Diego, USA	AB_314362
CD4, APC Fire 750, Clone RPAT4	BioLegend, San Diego, USA	AB_2629693
CD45, Alexa Fluor 700, Clone H130	BioLegend, San Diego, USA	AB_493761
CD45RA, BV605, Clone H100	BioLegend, San Diego, USA	AB_2563814
CD56, PerCP/Cy5.5, Clone HCD56	BioLegend, San Diego, USA	AB_893389
CD8, APC Fire 750, Clone SK1	BioLegend, San Diego, USA	AB_2572095
CXCR3, BV650, Clone G025H7	BioLegend, San Diego, USA	AB_2563870
CXCR5, PE/Dazzle 594, Clone J25204	BioLegend, San Diego, USA	AB_2563689
HLA-DR, BV785, Clone L243	BioLegend, San Diego, USA	AB_2563461
IgD, PE/Dazzle 594, Clone 1A6-2	BioLegend, San Diego, USA	AB_2616990
TCR ϒ/δ, APC, Clone 11F2	Miltenyi Biotec, Bergisch Gladbach, Germany	AB_2733463
TCR Vα7.2, BV785, Clone 3C10	BioLegend, San Diego, USA	AB_2566042
anti Biotin, PE, Clone REA746	Miltenyi Biotec, Bergisch Gladbach, Germany	AB_2661377
CCR6, PE/Dazzle 594, Clone G034E3	BioLegend, San Diego, USA	AB_2564233
CD11c, BV650, Clone Bu15	BioLegend, San Diego, USA	AB_2721551
CD127, APC, Clone A019D5	BioLegend, San Diego, USA	AB_10900804
CD14, BV605, Clone 63D3	BioLegend, San Diego, USA	AB_2716231
CD14, APC Fire 750, Clone M5E2	BioLegend, San Diego, USA	AB_2632659
CD16, PE/Dazzle 594, Clone 3G8	BioLegend, San Diego, USA	AB_2563639
CD161, BV605, Clone HP-3G10	BioLegend, San Diego, USA	AB_2563607
CD19, Alexa Fluor 700, Clone HIB19	BioLegend, San Diego, USA	AB_493751
CD20, FITC, Clone 2H7	BioLegend, San Diego, USA	AB_314252
CD25, BV421, Clone BC96	BioLegend, San Diego, USA	AB_11126749
CD27, PE/Dazzle 594, Clone O323	BioLegend, San Diego, USA	AB_2617005
CD27, BV785, Clone O323	BioLegend, San Diego, USA	AB_2562674

(*Continued on next page*)

CellPress
OPEN ACCESS

Continued

REAGENT or RESOURCE	SOURCE	IDENTIFIER
CD3, APC Fire 750, Clone UCHT1	BioLegend, San Diego, USA	AB_2629689
CD38, BV421, Clone HIT2	BioLegend, San Diego, USA	AB_10983072
CD4, PerCP/Cy5.5, Clone SK3	BioLegend, San Diego, USA	AB_1953236
CD45RA, BV650, Clone HI100	BioLegend, San Diego, USA	AB_2563653
CD56, BV421, Clone HCD56	BioLegend, San Diego, USA	AB_11218798
CD56, APC Fire 750, Clone 5.1H11	BioLegend, San Diego, USA	AB_2572104
CD8, BV785, Clone SK1	BioLegend, San Diego, USA	AB_2566202
CXCR3, BV650, Clone G025H7	BioLegend, San Diego, USA	AB_2563870
HLA-DR, BV650, Clone L243	BioLegend, San Diego, USA	AB_2563828
IgD, PerCP/Cy5.5, Clone W18340F	BioLegend, San Diego, USA	AB_2894457
TCR ϒ/δ, FITC, Clone B1	BioLegend, San Diego, USA	AB_1575108
Chemicals, peptides, and recombinant proteins		
BD FACS™ Lysing solution	BD Biosciences, Franklin Lakes, USA	Cat. # 347691
MACS® BSA Stock Solution	Miltenyi Biotec, Bergisch Gladbach, Germany	Cat. # 130-091-376
autoMACS® Rinsing Solution	Miltenyi Biotec, Bergisch Gladbach, Germany	Cat. # 130-091-222
CD19 CAR Detection Reagent, human	Miltenyi Biotec, Bergisch Gladbach, Germany	Cat. # 130-129-550; RRID: AB_2811310
Critical commercial assays		
Zombie Aqua™ Fixable Viability Kit	BioLegend, San Diego, USA	Cat. # 423101
BD Multitest™ 6-color TBNK	BD Biosciences, Franklin Lakes, USA	Cat. # 644611; RRID: AB_2870318
Software and algorithms		
FlowJoTM v 10.10.0	BD Biosciences, Franklin Lakes, USA	RRID:SCR_008520
Graphpad PRISM Software10	GraphPad Software, Boston, USA	RRID:SCR_002798
BD FACSDiva™ v9 Software	BD Biosciences, Franklin Lakes, USA	RRID:SCR_001456
BD FACSuite™ Clinical software v1.5	BD Biosciences, Franklin Lakes, USA	N/A
Other		
CD19 CAR Construct (KYV-101)	Kyverna Therapeutics, Emeryville, CA, USA	N/A
Precision Count Beads™	BioLegend, San Diego, USA	Cat. # 424902
BD Trucount™ Tubes	BD Biosciences, Franklin Lakes, USA	Cat. # 663028

EXPERIMENTAL MODEL AND STUDY PARTICIPANT DETAILS

Patients and clinical assessments

All three patients (2 female and 1 male) in this case series had severe, progressive generalized MG unresponsive to at least two standard immunomodulatory therapies including B-cell depleting antibodies. All cases were treated as individual treatment attempts (''individueller Heilversuch'') in accordance with the German legal framework and not within the context of a clinical trial.

Written informed consent was obtained in compliance with CARE guidelines and the Declaration of Helsinki, ensuring adherence to ethical standards and patient safety.[12–14] All patients provided informed consent for both the CAR T cell treatment and the publication of the results. All patients underwent standardized clinical evaluations, with disease severity assessed using the QMG score. Functional impairment in daily life was measured using the MG Activities of Daily Living scale (MG-ADL). Walking distance was determined through patients' reports.[37] Due to the small sample size of this case series, our data does not allow for the stratification of treatment outcomes by sex or gender.

For the patient who presented with concomitant rheumatoid arthritis (RA), RA activity was additionally monitored using the Disease Activity Score-28 (DAS-28) and Clinical Disease Activity Index (CDAI).[38]

Safety assessments

Patients were monitored daily for the development of CRS and immune effector cell–associated neurotoxicity syndrome (ICANS) during the first 10 days following anti-CD19 CAR T cell infusion. Both CRS and ICANS were evaluated in accordance with the consensus criteria established by the American Society for Transplantation and Cellular Therapy.[21]

Myelotoxicity was defined as grade III or IV neutropenia or leukocytopenia persisting for more than 28 days and was monitored prospectively using regular laboratory assessments, performed several times per week during the first 30 days, approximately biweekly between days 30 and 90, and at longer intervals thereafter. The monitoring comprised a complete blood count with differential, reticulocyte counts, C-reactive protein with optional ferritin, as well as lactate dehydrogenase, haptoglobin and interleukin-6.

CAR T cell manufacturing and treatment

A second-generation investigational CAR T cell product (KYV-101, Kyverna Therapeutics) was used.[39] Prior to infusion, all patients underwent lymphodepleting therapy with fludarabine (30 mg/m^2) and cyclophosphamide (300 mg/m^2) administered over three days (Table 1).[12–14]

METHOD DETAILS

Immunophenotyping for MG1 and MG3

Labeling of whole blood with fluorescent antibodies was performed according to standard protocols with staining panels adapted from Monaco et al.[40] Equal volumes of freshly drawn blood from EDTA-Tubes (Kabe Labortechnik, Nümbrecht, Germany) were mixed with Precision Count Beads (Cat. # 424902, BioLegend, San Diego, USA) to obtain absolute counts of cells. Red blood cells were lysed and samples fixed using BD FACS Lysing solution (Cat. # 347691, BD Biosciences, Franklin Lakes, USA). Samples were recorded on a BD FACSCelesta equipped with BD FACSDiva v9 software and analyzed with BD FlowJo v10.10.0. Antibodies used are found in the key resources table and gating strategies are summarized in (Figure S7).

Immunophenotyping and CAR T cell detection for MG2

For flow cytometry analysis, fresh whole blood samples were stained in PEB buffer, prepared by diluting MACS® BSA Stock Solution 1:20 with autoMACS Rinsing Solution (Miltenyi Biotec). Room temperature (RT) for incubation periods was defined as 19°C–25°C, RT for centrifugation steps was defined as 21°C. Centrifugation parameters were as follows: 300xg, 10 min, RT. Flow cytometry analyses were performed on a BD FACSCelesta Cell Analyzer (BD Biosciences). The data were analyzed with FlowJo software (BD Biosciences) and GraphPad Prism software (GraphPad Software).

For anti-CD19 CAR T cell detection, 100 µL whole blood sample was stained by adding 1:50 of the CD19 CAR Detection Reagent, anti-human, Biotin, REAfinity (Miltenyi Biotec), and after mixing incubated for 10 min in the dark at RT. Cells were washed by adding 1 mL of PEB buffer, and after mixing, centrifuged and supernatant aspirated completely. 1:50 Biotin Antibody, PE, REAfinity (Miltenyi Biotec) and antibodies with specificities for following cell surface proteins were added: CD45 (1:100), CD3 (1:200), CD4 (1:100), CD8 (1:100). For cell viability staining Zombie Aqua Fixable Viability Dye (1:2000; BioLegend) was added and incubated for 10 min in the dark at RT. Cells were washed by adding 1 mL of PEB buffer, and after mixing well, centrifuged. The supernatant was aspirated completely, and 2 mL of 1X BD FACS Lysing Solution 10X Concentrate (diluted in Aqua dest) was added. The sample was vortexed for 5 s and incubated for 7 min in the dark at RT. Cells were then centrifuged, the supernatant was aspirated completely, and 2 mL of PEB buffer was added. After mixing well, cells were centrifuged. This washing step was repeated. After the supernatant was aspirated completely, the cell pellet was dissolved in 100 µL PEB buffer, and 100 µL Precision Count Beads (BioLegend) were added.

For extracellular staining of immune cell population detection, 100 µL whole blood sample was stained by adding antibodies with specificities according to key resources table. For cell viability staining Zombie Aqua Fixable Viability Dye (1:2000; BioLegend) was added and incubated for 10 min in the dark at RT. The supernatant was aspirated completely, and 2 mL of 1X BD FACS Lysing Solution 10X Concentrate (diluted in Aqua dest) was added. The sample was vortexed for 5 s and incubated for 7 min in the dark at RT. Cells were then centrifuged, the supernatant was aspirated completely, and 2 mL of PEB buffer was added. After mixing well, cells were centrifuged. This washing step was repeated. After the supernatant was aspirated completely, the cell pellet was dissolved in 100 µL PEB buffer, and 100 µL Precision Count Beads (BioLegend) were added. The following gating strategy was used:

For all cells: Lymphocytes, Singlets (FSC-A/FSC-H), Dead cell exclusion (Zombie Aqua).

T-cells
- $CD3^+$, CAR^+, CD4/CD8 Crossgate
- $CD3^+$, CAR^-, CD4/CD8 Crossgate
  - CD4 Tregs: $CD25^{++}CD127^{-/low}$
  - Th1 ($CCR6^-/CXCR3^+$), Th2 ($CCR6^-/CXCR3^-$), Th17 (Crossgate $CCR6^+/CXCR3^-$, $CD161^+$)
  - $CD8^+$ → TE ($CD27^-/CD45RA^+$), EM ($CD27^-/CD45RA^-$), CM ($CD27^+/CD45RA^-$), naive ($CD27^+/CD45RA^+$)
  - $CD4^+$ → naive ($CD27^+/CD45RA^+$), TD ($CD27^-/CD45RA^+$)

γ/δ T-cells
- $CD3^+$, CAR^-, TCR γ/δ^+

NK cells
- $CD3^-$, CD56/CD16

B-cells
- Dump channel (CD3, CD56, CD16)

CellPress
OPEN ACCESS

- Neg population, CD19 → Naive (IgD^+, $CD27^-$), NSM (IgD^+, $CD27^+$), SM (IgD^-, $CD27^+$), Plasmacells/blasts (Antibody producing) → IgD^- → $CD38^{++}/CD27^{++}$

Data were analyzed using FlowJo v10.10, FlowJo LLC, 385 Williamson Way, Ashland, OR 97520, USA; GraphPad Prism version 10.0.0 for Windows, GraphPad Software, Boston, Massachusetts USA.

Detection and quantification of CAR T-cells for MG1 and MG3

Peripheral blood samples were collected in EDTA-anticoagulated tubes and processed promptly to ensure sample integrity. Samples were stained using the BD Multitest 6-Color TBNK Reagent (BD Biosciences, San Jose, CA, USA). Absolute cell counts were determined by combining stained samples with BD Trucount Tubes (BD Biosciences), following the manufacturer's instructions. Flow cytometric acquisition was performed on a BD FACSLyric flow cytometer (BD Biosciences). Data analysis, including gating strategies and subset classification, was conducted using BD FACSuite Clinical software v1.5 (BD Biosciences), adhering to the manufacturer's standard protocols. Lymphocyte populations were gated based on CD45 expression and forward/side scatter characteristics, with further discrimination of $CD3^+$ T-cells and their $CD4^+$ and $CD8^+$ subsets. Absolute counts were calculated using the Trucount bead-based method as per the manufacturer's guidelines. For specific detection of CAR T-cells, the CAR detection reagent (Miltenyi Biotec) in combination with anti-biotin antibody (Miltenyi Biotec) were utilized, enabling precise CAR T cell identification within the $CD3^+$ T cell compartment. All procedures were performed according to standardized protocols to ensure reproducibility and comparability of results across samples.

QUANTIFICATION AND STATISTICAL ANALYSIS

This study only provides descriptive data from individual patients without statistical analysis. Graphs were generated by GraphPad Prism v10.

Cell Reports Medicine, Volume *7*

Supplemental information

Long-term outcomes of anti-CD19 CAR T cell therapy in refractory myasthenia gravis: A case series

Tobias Hegelmaier, Jeremias Motte, Denise Walther, Eugen Feist, Alexander Duscha, Christiane Desel, Vaia Pappa, Martin Böttcher, Marvin Voigt, Melissa Sgodzai, Marwa Al-Dubai, Roland Schroers, Dominic Borie, Georg Schett, Dimitrios Mougiakakos, Ralf Gold, and Aiden Haghikia

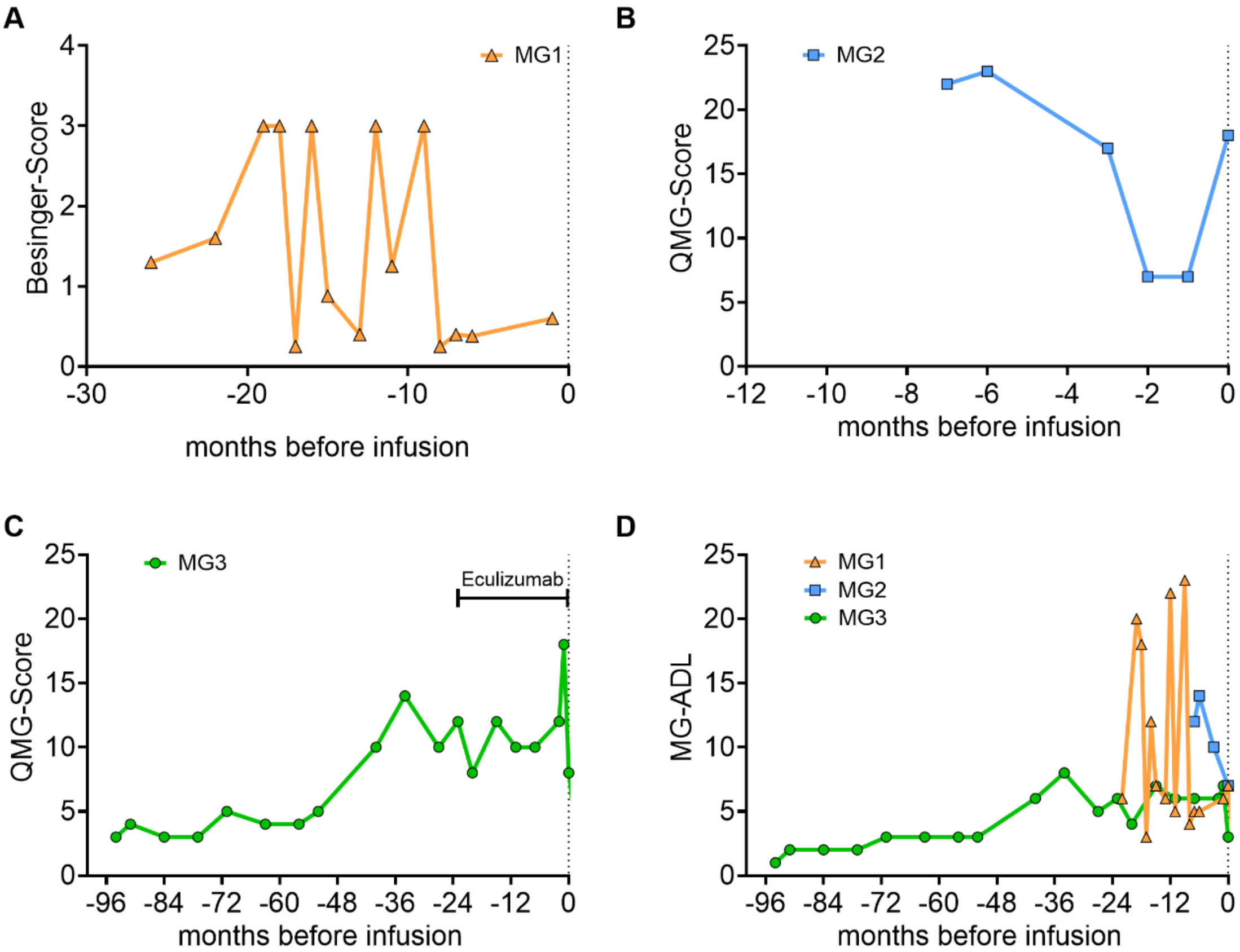

Figure S1. Baseline clinical charateristics prior to CAR T-cell infusion, Related to Tables 1-2. A: Besinger-Score of patient MG1 from March 2021 until CAR T-cell infusion in May 2023. B: Quantitative Myasthenia Gravis (QMG)-Score of patient MG2 from January 2023 until CAR T-cell infusion in August 2023. C: QMG-Score of patient MG3 from January 2016 until CAR T-cell infusion in November 2023. Eculizumab treatment from January 2022 with 900 mg escalating to 1200 mg in March 2022 until October 2023. D: Myasthenia Gravis Activities of Daily Living (MG-ADL) scores all three patients prior to CAR T-cell infusion.

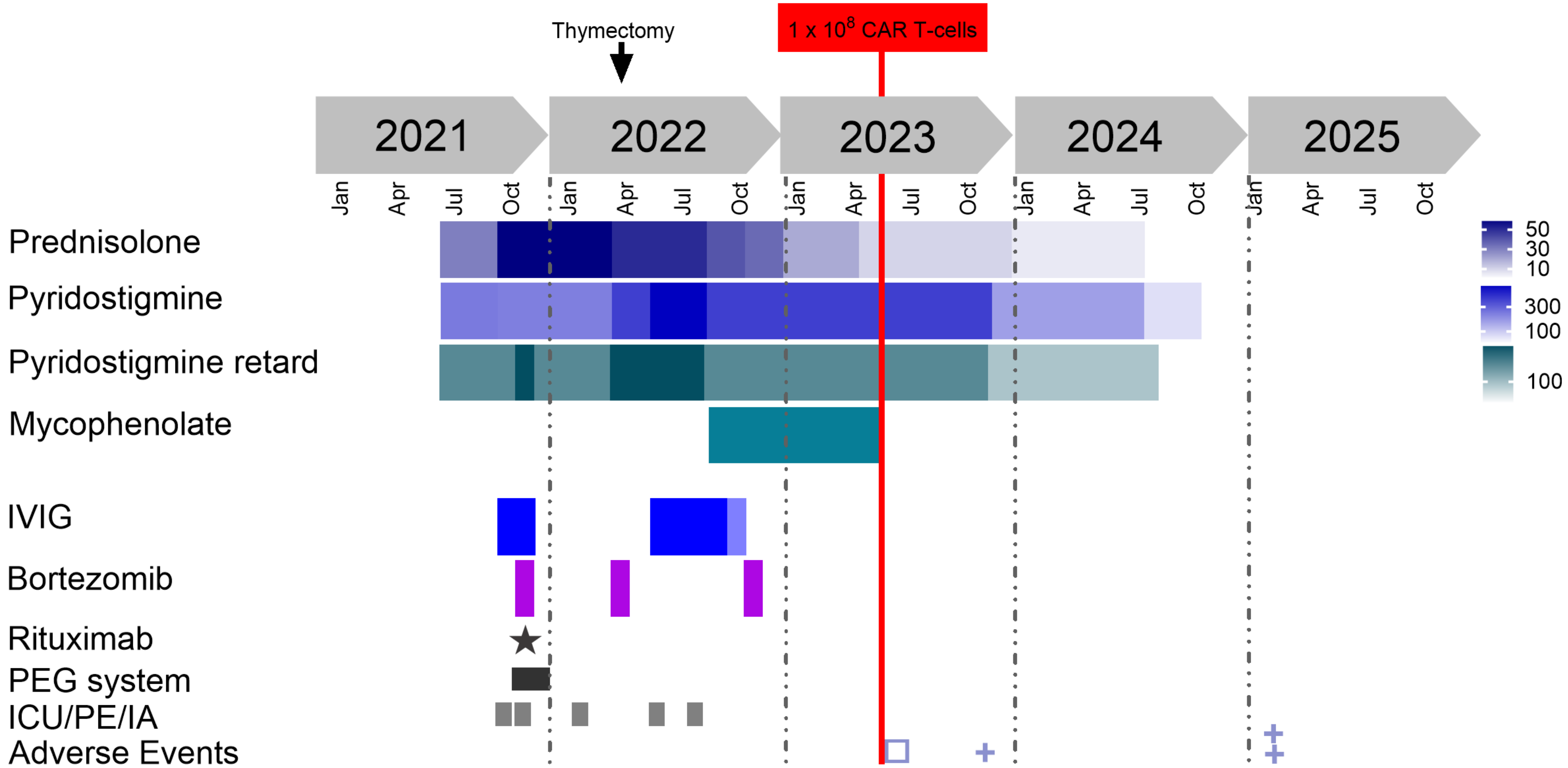

Figure S2. Treatment course of patient MG1, Related to Tables 1-2. Initial diagnosis in 2012. Doses depicted in [mg/day], unless stated otherwise. Mycophenolate: 1000 mg/day. Intravenous immune globulin (IVIG): 120 or 60 g; cumulative. Bortezomib: 4 doses each timepoint. Rituximab: 2 x 1000 mg. During acute myasthenic crisis Pyridostigmine perfusor. Intensive care unit (ICU) visits requiring intubation/ventilation. PE: plasmapheresis, IA: immunadsorption. Adverse events: Grade 1 trans-aminits (□), duration 19 days; SARS-CoV2 infection (+), duration 8 days; Pustulosis palmaris et plantaris (‡), duration 20 days; treated with Octenisept compresses (twice daily for 10–20 minutes) and local corticosteroid therapy (corticosteroid fat-based ointment, applied once daily for 4 hours).

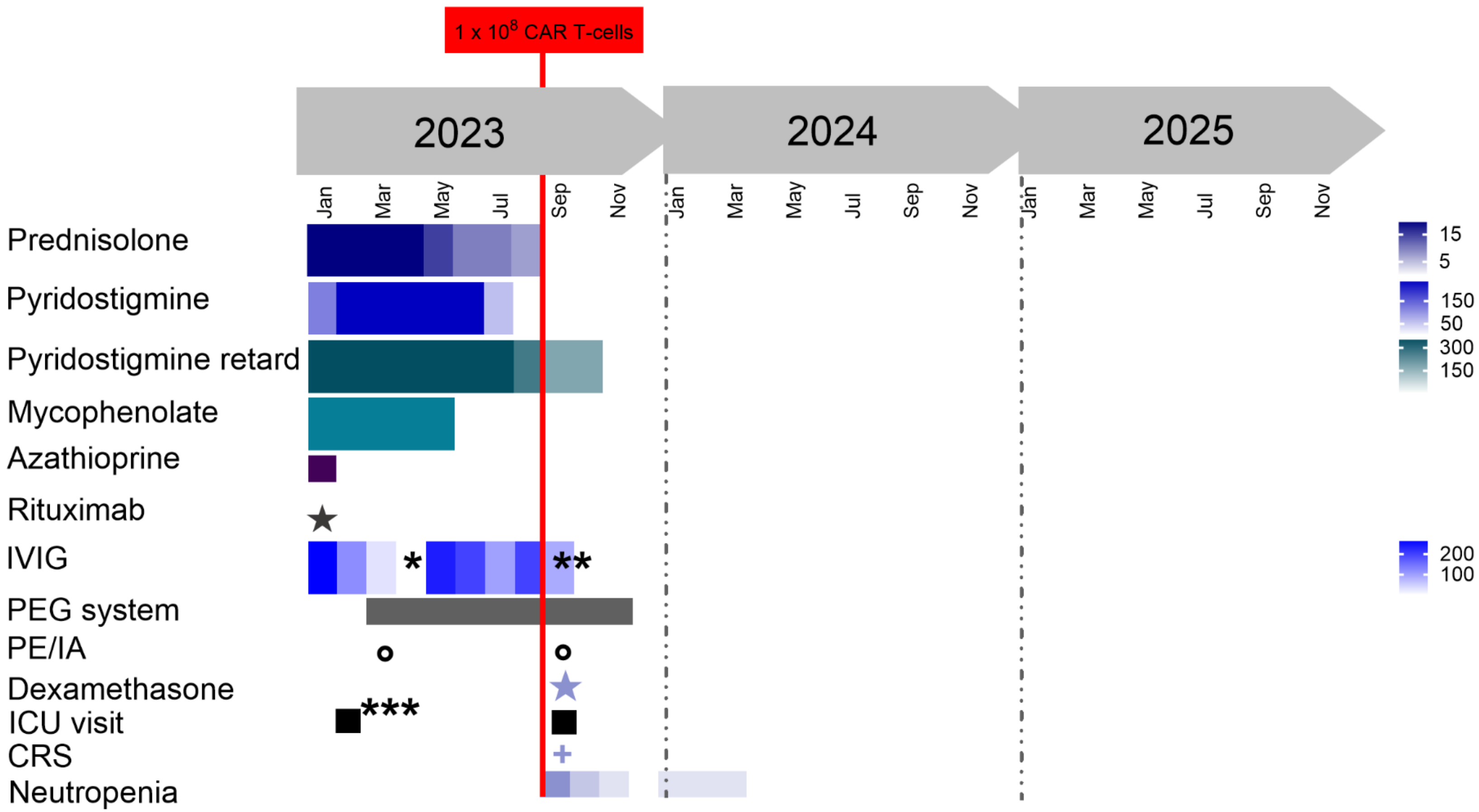

Figure S3. Treatment course of patient MG2, Related to Tables 1-2. Initial diagnosis in 2022. Doses depicted in [mg/day] unless stated otherwise. Prednisolone tapered in August 2023. During ICU visit in February 2023: 500 mg Methylprednisolone. Pyridostigmine: From July-Nov 2023 60mg daily only when necessary due to gastrointestinal adverse effects. Pyridostigmine retard: Tapered by the end of October 2023. Mycophenolate: 1000 mg. Azathioprine: Dose escalation aborted at 150mg due to side effects. Rituximab: 1 x 500 mg. Intravenous immune globulin (IVIG) in [g] cumulative dose, *April 2023: no information on IVIG dose available. ** September 2023: 90 g IVIG during ICU visit over three days. PEG: percutaneous endoscopic gastrostomy. PE: plasmapheresis, IA: immunadsorption. Dexamethasone 64 mg cumulative during ICU visit over three days. *** ICU visit for 4 days, cumulative 500mg Urbasone. ICU visits for close monitoring, not requiring intubation/ventilation. Without therapy since November 2023. Adverse events: CRS Grade 1 (+), duration 2 days; starting 6 days after CAR T-cell infusion. Grade IV neutropenia in September 2023 managed by a single dose of 32.000 IU G-CSF s.c.; recurring grade I neutropenia in 2024 lasting for 90 days without intervention.

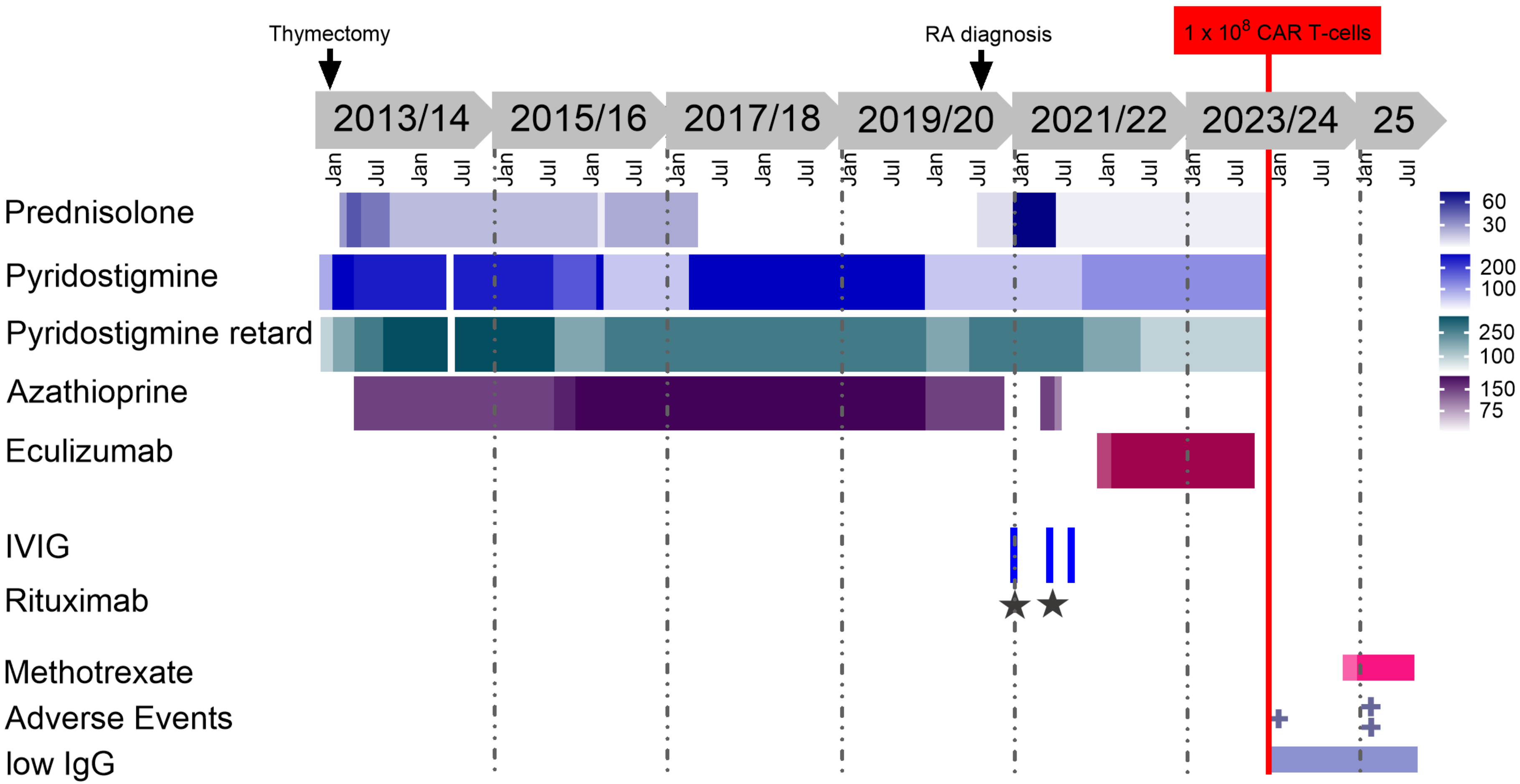

Figure S4. Treatment course of patient MG3, Related to Tables 1-2. Initial MG diagnosis in 2013, Rheumatiod arthritis (RA) diagnosis in 2020. Doses depicted in [mg/day], unless stated otherwise. Eculizumab: 900 or 1200 mg. Intravenous immune globulin (IVIG): 120 or 60 g cumulative dose. Rituximab: 500 mg. Methotrexate 10-15 mg subcutaneously. Prednisolone and Pyridostigmine were slowly tapered over the course of 5 weeks after CAR T-cell infusion. Adverse events: CRS Grade 1 (+), duration 1 day; upper respiratory tract infection (URTI) (‡). Low IgG persisting for the entire follow up period.

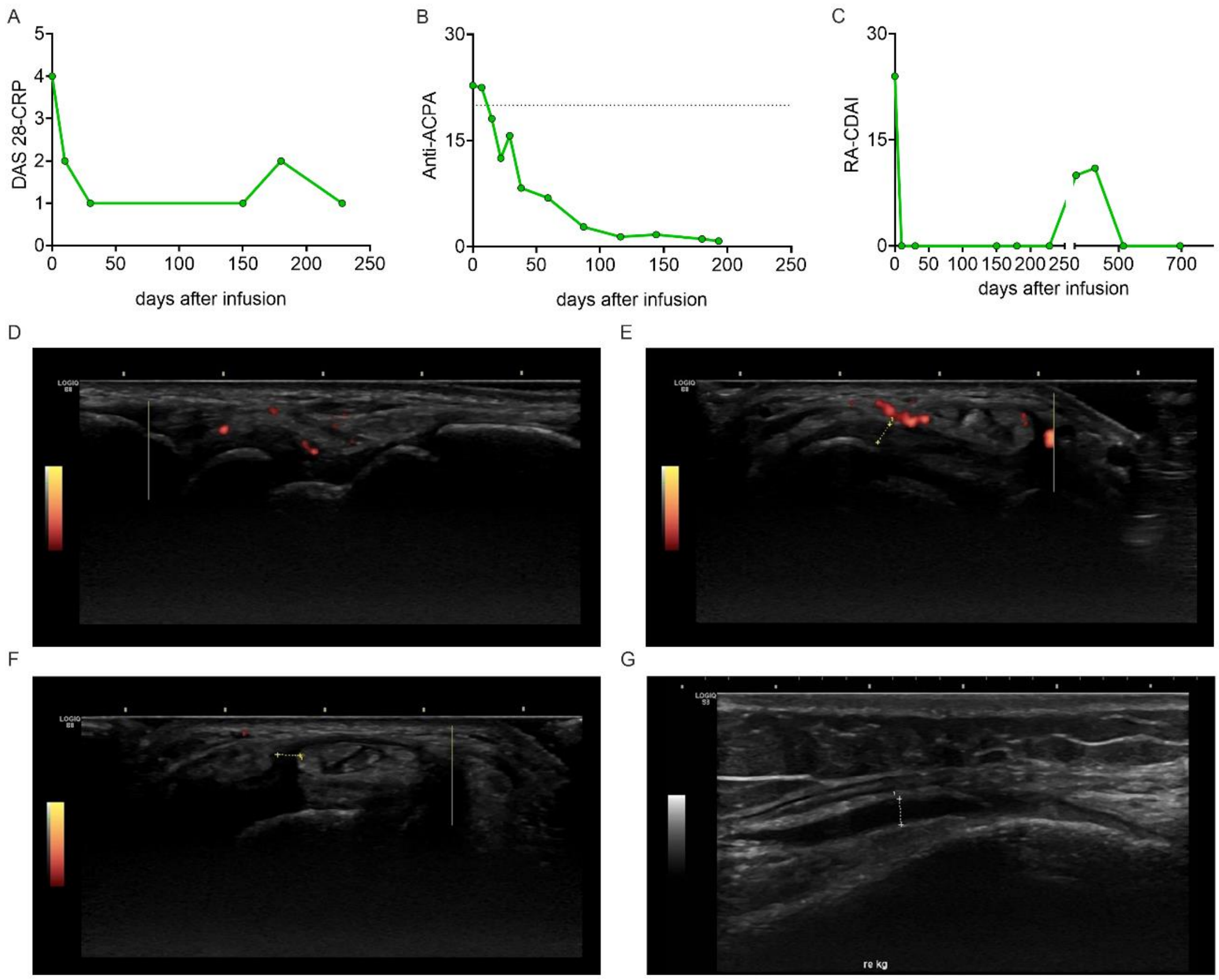

Figure S5. Serological markers of RA and ultrasound images following anti CD19 CAR T-cell therapy in patient MG3, Related to Figure 1. A: DAS-28 CRP Score. B: Anti-ACPA antibody levels. Dashed line depicts cut-off value of 20. C: RA-CDAI. D: Ultrasound image left wrist longitudinal with effusion and grade 1 synovitis. E: Ultrasound image left wrist transversal. F: Ultrasound image tenosynovitis right wrist transversal. G: Ultrasound image right knee longitudinal with mild effusion. RA = Rheumatoid Arthritis, DAS = Disease Activity Score, ACPA = Anti-Citrullinated Protein Antibodies, CDAI = Clinical Disease Activity Index.

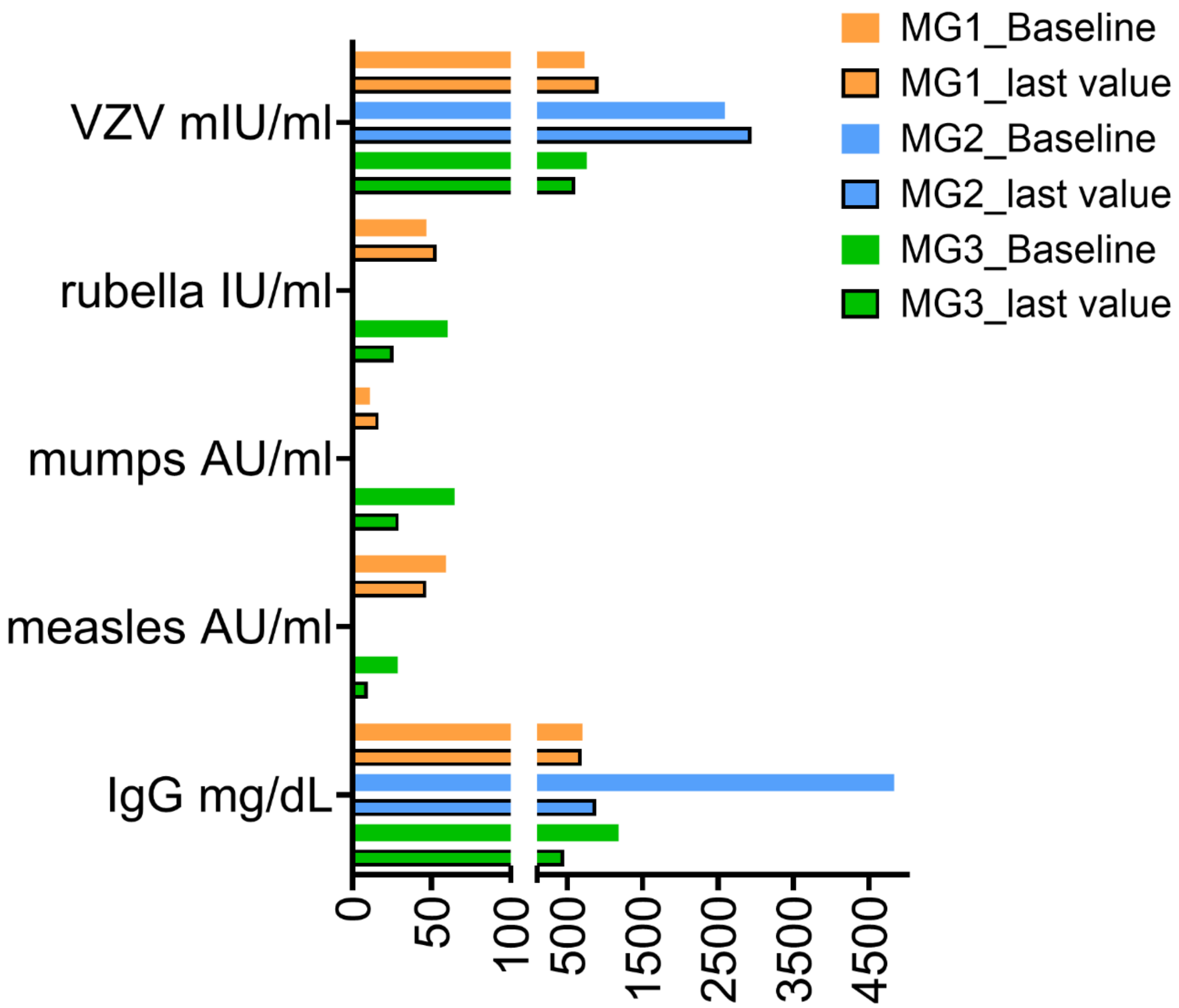

Figure S6. Vaccination titers remain stable after CAR T-cell infusion, Related to Figure 2. Baseline titers were determined before and last value refers to varying time points (60 – 200 days) after anti-CD19 CAR T-cell infusion. VZV = varicella zoster virus, MG1 = patient 1, MG2 = patient 2, MG3 = patient 3.

Panel 1 - CD4 T-cells

Cell type	Gating strategy	
**CD4+ T-cells (CD4)**	CD3$^+$ CD4$^+$	
T follicular helper (Tfh)		CXCR5$^+$
T regulatory cells (Tregs)		CXCR5$^-$ CD25^{high} CD127^{low}
T helper 1 (Th1)		CXCR5$^-$ CD127$^+$ CD25$^{-/low}$ CXCR3$^+$ CCR6$^-$
T helper 1/T helper 17 (Th1/Th17)		CXCR5$^-$ CD127$^+$ CD25$^{-/low}$ CXCR3$^+$ CCR6$^+$
T helper 17 (Th17)		CXCR5$^-$ CD127$^+$ CD25$^{-/low}$ CXCR3$^-$ CCR6$^+$
T helper 2 (Th2)		CXCR5$^-$ CD127$^+$ CD25$^{-/low}$ CXCR3$^-$ CCR6$^-$ CCR4$^+$
T CD4 terminal effector (CD4 TE)		CXCR5$^-$ CD127$^+$ CD25$^{-/low}$ CXCR3$^-$ CCR6$^-$ CCR4$^-$ CCR7$^-$ CD45RA^{low}
CD4 naïve		CXCR5$^-$ CD127$^+$ CD25$^{-/low}$ CXCR3$^-$ CCR6$^-$ CCR4$^-$ CCR7$^+$ CD45RA^{high}

Panel 2 - CD8, γ/δ and MAIT T-cells

Cell type	Gating strategy		
CD8+ T-cells (CD8)	CD3$^+$	TCR γ/δ$^-$	CD8$^+$ NOT CD161^{high} Vα7.2$^+$
γ/δ T cells		TCR γ/δ$^+$	Vd2$^+$ or Vd2$^-$
MAIT		TCR γ/δ$^-$ Vd2$^-$	Vα7.2$^+$ CD161^{high}
CD8 naïve		CD8$^+$ TCR γ/δ$^-$ Vd2$^-$ NOT MAIT	CCR7$^+$ CD45RA$^+$
CD8 central memory T-cells (CD8 CM)			CCR7$^+$ CD45RA$^-$
CD8 effector memory T-cells (CD8 EM)			CCR7$^-$ CD45RA$^-$
CD8 terminal effector T-cells (CD8 TE)			CCR7$^-$ CD45RA$^+$

PANEL 3 – B-cells and Progenitors

Cell type	Gating strategy		
CD34 Progenitor	CD45$^+$	CD34$^+$ CD45^{low}	
Naïve B-cells (CD19 naive)		CD19$^+$ CD34$^-$	CD27$^-$ IgD$^+$
Nonswitched memory B-cells (CD19 NSM)			CD27$^+$ IgD$^+$
Exhausted B-cells (CD19 EX)			CD27$^-$ IgD$^-$
Switched memory B-cells (CD19 SM)			CD27$^+$ IgD$^-$ CD38^{low}
Plasmablasts			CD27$^+$ IgD$^-$ CD38^{high}
Plasmacells		CD19$^-$ CD34$^-$	IgD$^+$

Panel 4 - Monocytes, DCs, NK cells, Low-density basophils

Cell type	Gating strategy		
NK cells	CD45$^+$ CD3$^-$ CD19$^-$		CD16$^{+/-}$ CD56$^{+/low}$ CD14$^-$ CD11c$^-$
Classic monocytes (Mono classic)			CD14$^+$ CD16$^-$
Intermediate monocytes (Mono inter)		CD11c$^+$	CD14$^+$ CD16$^+$
Nonclassic monocytes (Mono nonclassic)			CD14^{low} CD16$^+$
Myeloid dendritic cells (mDC)		CD16$^-$ CD56$^-$	HLA-DR$^+$ CD11c$^+$ CD123^{low}
Plasmacytoid dendritic cells (pDC)			HLA-DR$^+$ CD11c$^-$ CD123$^+$
Low-density basophils (LD basophils)			HLA-DR$^-$ CD123$^+$ CD11b$^+$

Figure S7. Gating strategies immunophenotyping for MG1 and MG3, Related to STAR Methods. Labelling of whole blood with fluorescent antibodies was performed according to standard protocols. Antibodies used are found in the key resource table.
